# Mental health in challenging situations: how experienced agency affects coping and mental distress

**DOI:** 10.3389/fpsyg.2026.1786909

**Published:** 2026-05-13

**Authors:** Luana Benz, Roland Pfister, Katharina A. Schwarz

**Affiliations:** 1 Department of Psychology III, University of Würzburg, Würzburg, Germany; 2 Institute of Psychology, Trier University, Trier, Germany; 3 Institute for Cognitive and Affective Neuroscience (ICAN), Trier University, Trier, Germany; 4 Department of Behavioural and Cognitive Sciences, University of Luxembourg, Esch-sur-Alzette, Luxembourg

**Keywords:** coping, distress, experienced agency, mental health, sense of agency

## Abstract

Mental health is becoming increasingly important in clinical settings and everyday situations alike. Here we address the role of experienced agency for mental health, i.e., the role of the experience of being in control and author of one's own actions and their consequences. The present study investigates how experience of agency is linked to mental health and coping in the face of personal and global challenges. Our data challenge a simple model of direct positive effects of agency experience for mental health for specific, challenging situations. Rather, they point toward a crucial impact of perceived responsibility that may influence when, in such situations, experiences of control affect mental health in a positive or negative manner. Interestingly, our data still confirm a generally positive association between trait assumptions of experienced agency and mental health. Stronger agency experience was further associated with more frequent use of control-related coping strategies. Exploratory analyses revealed a discrepancy between the actual use and the perceived effectiveness of coping strategies—a discrepancy that decreased with higher levels of experienced agency. Overall, the results highlight a close relationship between agency experience, mental health, and coping, while also pointing toward more complex mechanisms in specific challenging contexts. Interventions specifically aimed at strengthening agency experience may therefore represent a promising avenue for improving mental health especially when they employ measures to target the critical role of perceived responsibility.

## 1 Introduction

Today's world is characterized by increasing complexity, rapid change, and global challenges, as well as high demands on availability and performance in everyday life. Whether due to the impending effects of climate change, uncertainty caused by geopolitical conflicts, or high demands in the workplace, many people feel helpless, overwhelmed and lacking influence and control (World Health Organization, 2022). These circumstances can pose a major challenge and consequently they present a serious threat to mental health–understood as a state of wellbeing in which a person recognizes their own abilities, can cope with the normal stresses of life, can work productively, and is able to contribute to the community (Hosman et al., 2004; World Health Organization, 2001, 2022; Keyes, 2002).

### 1.1 Agency experience and mental health

Importantly, experienced agency, or sense of agency, does not reflect one's objective agency, i.e., objective causal relationships between own actions and effects, but relies on subjective perception and interpretation (Schwarz, under review). Agency experience encompasses two central, aspects that, while being closely related, are distinct in emphasis and do not always align: Firstly, agency experience refers to the experience of control, i.e., the mental representation of controlling environmental changes through action (“Am I in control?”), and secondly, authorship attribution, i.e., the mental representation of being the cause for environmental changes (“Was that me?”) (Schwarz et al., 2025, under review; see also Nadelhoffer et al., 2014; Nahmias, 2005). Because the majority of empirical evidence and theorizing on experienced agency has focused on control experience (e.g., Haggard and Tsakiris, 2009; Haggard, 2017; Schwarz et al., 2025), authorship attribution is a surprisingly overlooked, but crucial element of agency experience, distinguishing between self- and not-self produced environmental changes (e.g., Gallagher, 2000; Schwarz et al., 2023, 2025; Weller et al., 2017). Indeed, experienced agency may likely arise when control experience is low, but authorship attribution high (e.g., when inadvertently knocking a glass of water off a table). Recent empirical evidence indicates that control experience is based largely on comparisons between behavioral goals and outcome (Kumar and Srinivasan, 2014; Schreiner et al., 2025); however, if one's own contribution becomes negligible and, thus authorship attribution suffers, experienced agency breaks down even if goal attainment is at an almost perfect level (e.g., Ueda et al., 2021). Differences in conceptualizations of both aspects are also visible with regard to their association with related concepts: Perceived responsibility has often been discussed as a direct downstream consequence of experienced agency (e.g., Weller et al., 2020). However, closer analyses reveal that whereas perceived authorship is very strongly associated to perceived responsibility with only nuanced differences (explained variance of 60 to 80%, see Schwarz et al., 2023), it is less strongly associated with perceived control (explained variance of 30 to 40%, see Reis et al., 2022). Agency experience can be further broken down into explicit agency judgments and implicit agency intuition (Haggard and Tsakiris, 2009; Moore, 2016; Schwarz, under review; Synofzik et al., 2008): While explicit agency judgments refer to the conscious, conceptual attribution of authorship and control, implicit agency intuition describes the intuitive, in-the-moment experience of authorship and control over an action currently being performed.

Agency experience has proven to be a crucial construct in understanding concepts such as responsibility, voluntariness, and free will, and it plays a central role in social contexts—for instance, when it comes to accountability or the experience of guilt (Frith, 2014; Haggard and Tsakiris, 2009; Moore, 2016). Indeed, the association of perceived responsibility and authorship assumption is so strong that agency experience is often evaluated via perceived responsibility (for an analysis of the association, see, e.g., Schwarz et al., 2023). Moreover, it affects action choice, thus influencing behavioral decisions (e.g., Karsh and Eitam, 2015; Hemed et al., 2022; Schwarz et al., 2022, 2025; Reis et al., 2023). Importantly, research also indicates that agency experience contributes significantly to mental health and psychological wellbeing (Moore, 2016): It has long been recognized that the perception of control and influence constitutes a fundamental psychological need (e.g., Grawe, 2017; Skinner, 2007; White, 1959). More recently, perceived control is increasingly acknowledged as a critical factor in shaping mental health and wellbeing, as well as motivation and drive (e.g., Adler, 2011; Gallagher et al., 2014; Ly et al., 2019; Skinner, 2007).

Research in clinical psychology further indicates that an altered agency experience is associated with various mental disorders, including depression, anxiety disorders, and schizophrenia (Ly et al., 2019; Moore, 2016). A reduced experience of agency has repeatedly been linked to depression, as a perceived global loss of control can lead to passivity and low motivation, reinforcing depressive symptoms in a downward spiral (Liu et al., 2015; Ly et al., 2019; Slaby et al., 2013; Vogel et al., 2024). This is consistent with the concept of learned helplessness, which suggests that repeated exposure to uncontrollable situations can result in emotional distress, passivity, and ultimately the development of depressive symptoms (Ly et al., 2019; Maier and Seligman, 1976; Miller and Seligman, 1975; Mineka and Hendersen, 1985). A lack of perceived control also plays a central role in anxiety disorders and is considered a psychological vulnerability factor: early experiences of loss of control can promote the development of a cognitive style in which future events are interpreted as uncontrollable (Bargai et al., 2007; Chorpita and Barlow, 1998; Gallagher et al., 2014; Huys and Dayan, 2009; Liu et al., 2015). In individuals with existing anxiety disorders, cognitive-affective processes contribute to the maintenance of symptoms, as beliefs about the uncontrollability of anxiety and external events can further exacerbate the condition (Gallagher et al., 2014). In schizophrenia, disturbances in agency experience are discussed as a potential underlying cause of positive symptoms such as hallucinations or delusions of external control, which are thought to result from impaired sensorimotor processes and disrupted self-attribution processes (Jeannerod, 2009; Moore, 2016).

Given these associations, the concept of agency experience is gaining increasing relevance in clinical psychology. Longitudinal studies indicate that increases in agency experience pave the way for improved therapeutic outcomes and greater psychological wellbeing (Adler, 2011). Accordingly, therapeutic interventions—particularly in the treatment of depression and anxiety disorders— may benefit from targeting perceived control as a central mechanism (Gallagher et al., 2014; Ly et al., 2019; Slaby et al., 2013).

A growing body of research has identified a positive association between a heightened sense of control (as an aspect of experienced agency) and better subclinical mental health outcomes across diverse settings (e.g., Endler et al., 2000; Krause et al., 1992; Lawrance et al., 2022; McElwee and Brittain, 2009). For instance, higher levels of perceived control while solving anagram tasks were shown to be associated with better performance, reduced anxiety, and less psychological stress—regardless of the objectively given control (Endler et al., 2000). Similarly, other studies have found that individuals tend to be more optimistic about personal than global future events, a pattern attributed to a stronger sense of subjective control, which is, in turn, associated with greater wellbeing and optimism (McElwee and Brittain, 2009; Weinstein, 1980; Wenglert and Rosén, 2000). Research in the context of volunteer work further supports this relationship, showing that higher levels of perceived control and self-efficacy^1^ are associated with improved mental wellbeing and reduced depressive symptoms (Brown et al., 2012; Krause et al., 1992). Recent research also highlights the significance of experienced agency in the face of global challenges such as climate change and the COVID-19 pandemic —contexts that are often experienced as uncontrollable, mentally distressing, and marked by negative emotional responses (Lawrance et al., 2022; Yatirajula et al., 2023). However, higher perceived agency and actionability in response to such crises were associated with better mental health outcomes, suggesting that strengthening agency experience may enhance psychological resilience (Asbrand et al., 2024).

Nevertheless, altogether, findings remain somewhat mixed, with some studies reporting only weak or inconsistent associations between agency experience and mental wellbeing (e.g., Lawrance et al., 2022; Mert and Wang, 2023). Adding further complexity, in addition to affecting mental health directly, agency experience also may impact mental wellbeing by affecting how individuals cope, i.e., which coping strategies they choose, to deal with stressful, challenging situations.

### 1.2 Coping strategies

Coping strategies are a major factor in deciding how stressful situations affect mental health (Dijkstra and Homan, 2016). Coping refers to all cognitive and behavioral efforts used to manage internal or external demands that are appraised as stressful or exceeding one's resources in the interaction with the environment (Folkman et al., 1986).

Coping strategies differ with regard to their target (e.g., problem-focused vs. emotion-focused strategies; Conway and Terry, 1992; Endler et al., 2000) and their level of activity (active vs. passive strategies; Dijkstra and Homan, 2016). Particularly active coping strategies, such as direct confrontation are associated with greater coping success and better mental wellbeing compared to passive, avoidant approaches (e.g., Chao, 2011; Koeske et al., 1993). In these instances, perceived control has been suggested to serve as a mediating mechanism (Asbrand et al., 2024; Dijkstra and Homan, 2016; Litman and Lunsford, 2009): Individuals with a stronger agency experience might be more likely to perceive stressful situations as controllable and use active and problem-oriented coping strategies (Asbrand et al., 2024). In contrast, lower agency may be associated with reduced perceived control and thereby increase the likelihood of passive or avoidant coping responses. Through these pathways, agency experience may indirectly influence mental wellbeing by affecting coping behavior (Dijkstra and Homan, 2016; Litman and Lunsford, 2009).

### 1.3 Study aim and hypotheses

The present study aims to clarify the complex relationship between experienced agency and mental health, with a specific focus on coping strategies. More precisely, it aims at bridging prevailing gaps and potentially providing explanations for previous mixed results regarding the association of agency experience and mental wellbeing, by expanding on both factors, i.e., on agency measurements (trait and situational; control and authorship/responsibility as the driving subcomponents of agency experience) and on situational factors that may affect this association. For example, previous literature suggests that globally compared to personally challenging situations should result in smaller agency experience, as exclusivity assumptions are a strong driver of agency experience (see, e.g., Schwarz et al., 2023; Wegner, 2003). However, it remains unclear how this, in turn, might impact mental health. The present study aims to provide first exploratory glimpses into these questions.

Moreover, the study aims to further clarify how experienced agency may relate to potential coping strategies as a further influence on individual wellbeing (see, e.g., Asbrand et al., 2024). Higher levels of experienced agency may foster the use of more active, control-related coping strategies, which are considered more effective than passive and avoidant approaches. Exploratorily, we aimed at elaborating whether experienced agency may not only affect the coping strategies they choose as particularly helpful, but also whether individuals engage in coping strategies that they perceive as helpful in stressful situations, thereby examining the alignment between perceived helpfulness and actual use of coping strategies.

Ultimately, we hope to derive implications that can lay the groundwork for future interventions aimed at protecting and promoting mental health. To this end, we conducted a survey of 241 participants to analyse associations between general mental health and trait sense of agency as well as the impact of controllability and, as a direct downstream consequence of authorship experience, responsibility perception on mental health in challenging situations.

We expect that *(1) a stronger trait sense of agency*^2^ *is related to better self-reported mental health and fewer mental health problems*. In addition, we further expect that *(2) in specific challenging situations, negative effects on mental health and emotions will be less severe when participants still perceive some degree of control over the situation*. Moreover, based on previous research, we expect an association of agency experience and the use of coping strategies, in that *(3) a more pronounced trait sense of agency is associated with more frequent use of control-related coping strategies*. Finally, we hypothesize that *(4) control-related coping strategies are perceived as more helpful compared to other strategies*.

## 2 Materials and methods

The preregistration of this study is available on the Open Science Framework (OSF): osf.io/4zjvd/. All data files and analysis scripts are available on osf.io/e8r5h/.

### 2.1 Participants

A power analysis was conducted using the *pwr* package in R to determine the necessary sample size. We aimed for a power of 0.80 to detect correlations of *r* = 0.20, given a significance level of α = 0.05, resulting in a minimum of 194 participants. To account for potential exclusions, we aimed for a target sample size of 110% this number (213 participants).

Data was collected online and occurred in two phases, first at Julius-Maximilians-University Würzburg and subsequently at Trier University. Recruitment and registration were initially managed via Sona Systems and later through Prolific. Participants received either monetary compensation (€5) or course credit toward their psychology degree for their involvement.

In total, 241 individuals participated in the present study. Due to incomplete datasets, 14 datasets were excluded from analysis. Additionally, 15 further participants were excluded because they completed the questionnaire in under 10 min, suggesting low diligence (estimated completion time, supported by pilot data, was 20-30 min). Consequently, 212 participant datasets remained for data analysis, which corresponds to a statistical power of 83.6% for *r* = 0.2. Of these participants, 134 (64.3%) were female, 75 (34.4%) were male, and one person identified as non-binary. The remaining two individuals did not provide information about their gender. Participant ages ranged from 19 to 68 years, with an average age of 26.99 years. Further socio-demographic information (such as level of education, household income, household members, country and region) can be found in the Supplement.

### 2.2 Stimuli and apparatus

The present study was conducted in German language online on PCs or tablets and took approximately 30 min to complete. It was created and administered using SoSci Survey.

In addition to questions formulated based on previous research, the online study contained two previously established questionnaires: the German version of the Sense of Agency Scale (SoAS) to measure agency experience as a trait (Bart et al., 2023, based on Tapal et al., 2017), and the German General Self-Efficacy Scale (SE Scale) to assess self-efficacy as a related but distinct construct (Schwarzer and Jerusalem, 1999).

### 2.3 Procedure

**Figure 1:**
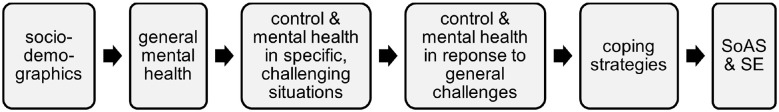
Study components. For detailed explanation, see the text, SoAS = Sense of Agency Scale (Bart et al., 2023; Tapal et al., 2017); SE = General Self-Efficacy Scale (Schwarzer and Jerusalem, 1999).

The study consisted of several consecutive parts (see Figure 1). In addition to the following descriptions, the exact wording and order of texts, questions, and items are provided in the Supplement.

The first pages of the online study provided information about the study and data protection. Informed consent was required before participation could proceed; if consent was not granted, the study was automatically terminated. Otherwise, participants answered several questions regarding socio-demographic information, including age, gender, education level, income, household members, country, and region.

Subsequently, participants were asked about their prior experiences with the topic of mental health in order to examine potential associations between mental health, SoA, and coping strategies. After a brief definition of the term “mental health” according to the World Health Organization (2001), an initial filter question assessed whether participants had previously engaged with the topic. If participants agreed, a multiple-choice filter question with an additional open-response option captured the nature of their previous contact (personal mental health/ social environment/ other). If participants indicated experiences through their social environment, follow-up questions assessed whether problems or the use of support services had occurred within their close or extended social network. If participants indicated personal experience with mental health as a point of contact, they were asked to specify their personal experience (interest/ subclinical problems/ mental disorders). In cases where subclinical symptoms or mental disorders were selected, participants were prompted to specify them in an open-ended text box. In addition, respondents were asked about their use of professional assistance services, and-if they had used such services-, they were asked to specify the type of assistance in an open-ended question. Regardless of previous responses, all participants were asked to evaluate their current mental health through a direct question (“How would you rate your current mental health?”) using a slider scale ranging from “very poor” to “very good.” This single-item measure was intended to capture participants' global self-evaluation of their current mental health. Single-item self-rated mental health measures are widely used in survey research and have been shown to correlate with more comprehensive mental health assessments and related life events, supporting their validity as global indicators of perceived mental wellbeing (Ahmad et al., 2014).

The following part of the study, focused on the perception of and responses to challenging situations. For this purpose, participants were presented with four short descriptions of specific challenging scenarios in randomized order. Two of these referred to global stressors (climate crisis, war), while the other two addressed personally distressing situations (conflict, stress). Each scenario was rated by participants using a slider scale ranging from “not at all” to “very strongly,” assessing the intensity of various emotions elicited (anger, fear, helplessness, guilt, shame, frustration, sadness, hope, and other [open-ended input]), perceived controllability by oneself and by others, sense of responsibility, degree of strain and finally the perceived impact on mental health (from “very positive impact” to “very negative impact”)^3^. Please note that perceived control and perceived responsibility were chosen as reflections of the two subcomponents of agency experience, i.e., perceived control and authorship attribution. Based on previous experience, we suspected that direct authorship evaluations might be semantically confusing for participants in an imagined scenario; therefore, we opted for perceived responsibility as a sufficiently close proxy (e.g., Schwarz et al., 2023; see also Reis et al., 2022; Schwarz et al., 2019; Weller et al., 2017).

After a prompt encouraging participants to take a short break, similar questions were presented once again—this time referring to the general perception of distressing situations, independent of specific events. Participants were asked to evaluate, separately for global and personal challenges, the emotions elicited, perceived controllability (by themselves and by others), the degree of strain and the perceived impact on mental health. Additionally, the frequency of feeling overwhelmed by and thinking about global and personal stressors was assessed using a scale ranging from “very rarely” to “several times a day.” Slider scales were again used as the response format. The only exception concerned the emotion-related questions: pilot studies had shown that rating the intensity of emotions without reference to concrete situations was difficult for participants. As a result, instead of intensity ratings, participants were asked to indicate the general presence of emotions using a simple multiple-choice format.

The subsequent section addressed the use and perceived helpfulness of coping strategies. Participants were first given a brief definition of the term (based on Folkman et al., 1986), followed by concise descriptions of eight overarching coping strategies, including Focus on Taking Actions, Social Support, Knowledge, Professional Support, Acceptance, Optimism, Avoidance and Distraction and Giving Up Responsibility (for detailed descriptions, see Supplement). These strategies were then presented in randomized order and participants rated both their frequency of use (“never” – “almost always”) and their perceived helpfulness (“not helpful at all”–“very helpful”) on slider scales. Subsequently, participants ranked the coping strategies using a drag-and-drop format—once according to frequency of use and once according to perceived helpfulness. The order of these ranking tasks (frequency vs. helpfulness) was randomized across participants. Finally, open-ended text fields invited participants to indicate additional coping strategies they frequently used or perceived as particularly helpful.

In addition, participants completed the German version of the Sense of Agency Scale (Bart et al., 2023, based on Tapal et al., 2017) as well as the General Self-Efficacy Scale (Schwarzer and Jerusalem, 1999). The items within each scale were presented in randomized order.

At the end of the survey, a text field was provided for comments or feedback. To ensure data quality, participants were asked to confirm that they had followed the instructions and given the study their full attention. Finally, a personal code was created by each participant for obtaining their compensation.

## 3 Results

### 3.1 Trait agency experience and mental health (hypothesis 1)

#### 3.1.1 Analysis plan

Hypothesis 1 stated that a strong trait sense of agency, measured by the Sense of Agency Scale (Bart et al., 2023), would be associated with better self-rated mental health and fewer self-reported mental health problems. To analyze the relationship between trait sense of agency and mental health, a Pearson correlation was first conducted between participants' self-assessment of their mental health (ranging from “very poor” to “very good”) and their mean scores on the Sense of Agency Scale. In addition, it was examined whether there were mean differences in trait sense of agency between individuals reporting no mental health problems, subclinical mental health problems, and diagnosed psychological disorders. To this end, a one-way analysis of variance (ANOVA; factor: self-reported mental health status [diagnosed problems vs. subclinical or undiagnosed problems vs. no problems]; dependent variable: SoAS) was conducted, followed by pairwise independent samples *t*-tests (two-tailed).

#### 3.1.2 Key findings

**Figure 2:**
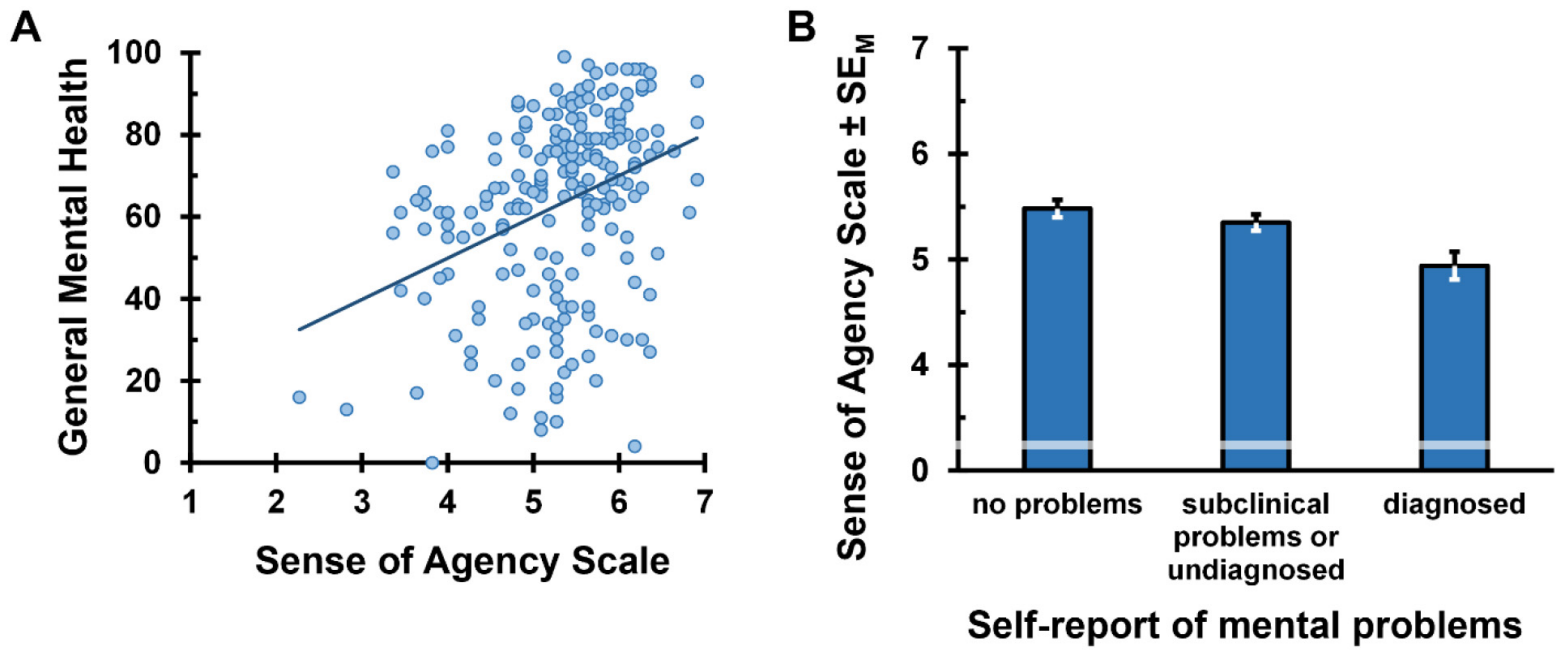
Relationship between agency experience and self-reported mental health (higher scores indicate better mental health). **(A)** Relation of self-rated mental health and Sense of Agency Scale scores. **(B)** Mean Sense of Agency Scale scores across different mental health status groups.

Self-rated overall mental health and the mean SoAS score were positively correlated, *r* = 0.35, *p* < 0.001 (see Figure 2A)^4^. Thus, a higher trait sense of agency was associated with better mental health.

The subsequent ANOVA showed significant differences in mean SoAS scores across self-reported mental health groups, *F*_(2, 209)_ = 7.94, *p* < 0.001, $\eta_{\text{p}}^{2}$ = 0.07 (see Figure 2B), that were mainly driven by a significant difference between participants with diagnosed mental health problems and both other groups, *M*_*without*_ = 5.5, *n*_*without*_ = 84, *M*_*subclinical*_ = 5.3, *n*_*subclinical*_ = 77, *M*_*diagnosed*_ = 4.9, *n*_*diagnosed*_ = 51; without problems vs. diagnosed problems: *t*(133) = 3.68, *p* < 0.001, *d* = 0.65; subclinical problems vs. diagnosed problems: *t*(126) = 2.88, *p* = 0.005, *d* = 0.52. In contrast, trait sense of agency between individuals with subclinical or undiagnosed mental health problems and those without any reported mental health issues did not differ significantly, *t*(159) = 1.17, *p* = 0.245, *d* = 0.18. Thus, as expected, participants with clinically relevant psychological disorders scored significantly lower on the SoAS compared to those with no or only subclinical mental health problems.

### 3.2 Agency experience and mental health in challenging situations (hypothesis 2)

#### 3.2.1 Analysis plan

We expected the negative effects on mental health and emotions in specific, challenging situations to be less pronounced when participants perceive a higher level of control over the situation.

First, as preliminary analyses, repeated-measures ANOVAs were conducted to test for differences in perceived control, perceived responsibility, perceived strain, and effects on mental health across the four specific situations (stress, conflict, war, climate change). Building on their results, *post hoc* paired-samples *t*-tests were conducted to further examine the differences in the dependent variables between the global and personal situations.

To test the main hypothesis, the association between perceived control and effects on mental health in the specific situations was first examined (Hypothesis 2a), followed by an examination of the association between perceived control and the intensity of negative emotions (Hypothesis 2b).

For this purpose, a hierarchical regression across all situations was conducted, sequentially entering situation (personal/global), perceived control, perceived responsibility, and perceived strain as predictors, with perceived impact on mental health serving as the criterion. Please note that for these analyses higher scores for “impact on mental health” indicate a stronger negative impact on mental health as this reflected the rating scale the participants answered (see Methods). To gain a better understanding of the influence of the situation, separate multiple hierarchical regressions were conducted for the personally and the globally challenging situations. In both models, perceived control, perceived responsibility, and psychological strain served as predictors, while perceived impact on mental health functioned as the dependent variable (details can be found in the Supplement).

To further examine the impact of perceived control and responsibility on mental health, exploratory mediation analyses were conducted for the different situations using the R package “mediation”. Perceived control served as the independent variable, mental health as the dependent variable, and perceived responsibility as the mediator (e.g., Zhao et al., 2010). For the mediation analysis, the impact on mental health ratings were reverse-coded, whereby higher values represent a more positive impact on mental health.

To address the second part of the hypothesis (the association between perceived control and negative emotions), a preliminary analysis was conducted to determine whether significant differences existed in emotion ratings. For this purpose, a 4x8 repeated-measures ANOVA with the factors situation (conflict, stress, climate, war) and emotion (anger, fear, helplessness, guilt, shame, frustration, sadness, hope) was performed.

To address the main hypothesis—namely, the question of the association between perceived control and negative emotions—a hierarchical multiple regression was conducted. The dependent variable was the mean rating of the various negative emotions (anger, fear, helplessness, guilt, shame, frustration, sadness), hereafter referred to as “negative emotion.” In addition to situation and perceived control, perceived responsibility and psychological strain were again included as exploratory predictors.

For further exploration, separate multiple hierarchical regressions were conducted for each negative emotion, again including situation, perceived control, and responsibility as predictors. The dependent variable in each model was the intensity of the respective negative emotion (fear, anger, shame, guilt, frustration, sadness, helplessness) (for details see Supplement).

#### 3.2.2 Key findings

**Figure 3:**
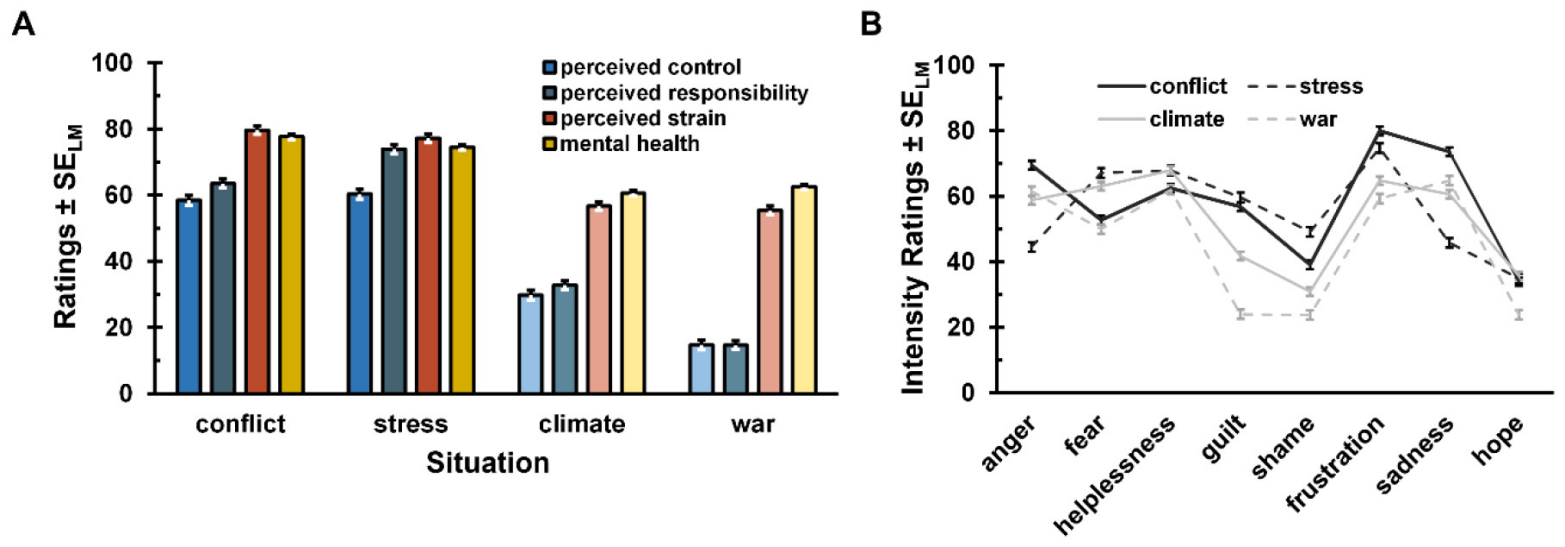
Perception of and emotional responses to different challenging situations. **(A)** Perceived control, responsibility, strain, and impact on mental health across different situations. Please note that higher scores in “impact on mental health” represent a stronger negative impact on mental health for these ratings, reflecting the participants' actual rating scale (see Methods). **(B)** Emotional intensity across different situations. Darker colors indicate personally challenging situations, lighter colors indicate globally challenging situations. Error bars indicate within-subjects standard errors according to Loftus and Masson (1994).

Figure 3A shows that participants' response patterns regarding perceived control, responsibility, strain, and mental health were similar within a given situation type-that is, within personal situations (e.g., conflict, stress) and within global situations (e.g., climate change, war). However, between situation types (personal vs. global) distinct patterns were observed. The ANOVAs of the preliminary analyses consistently revealed significant main effects of the situation: perceived control, *F*_(3, 633)_ = 229.41, *p* < 0.001, η_p_^2^ =0.52, ε = 0.93 (GG-corrected); perceived responsibility, *F*_(3, 627)_ = 408.27, *p* < 0.001, η_p_^2^ = 0.66, ε = 0.92 (GG-corrected); strain, *F*_(3, 630)_ = 97.00, *p* < 0.001, η_p_^2^ = 0.32, ε = 0.82 (GG-corrected); and mental health, *F*_(3, 621)_ = 101.21, *p* < 0.001, η_p_^2^ = 0.33, ε = 0.90 (GG-corrected). To accommodate for these patterns, we introduced the factor situation type for following analyses.

Subsequent *t*-tests conducted to further examine differences between personal and global situations revealed significant effects across all variables (perceived control, perceived responsibility, strain, and mental health; all *p* < 0.001), with consistently large effect sizes. Perceived control was significantly higher in personal situations compared to global situations, *M*_Control_personal_ = 59.4, *M*_Control_global_ = 22.2, *t*(211) = 22.46, *p* < 0.001, *d*_z_ = 1.54. A similar pattern emerged for perceived responsibility, *M*_Responsibility_personal =_ 68.7, *M*_Responsibility_global_ = 23.6, *t*(211) = 30.14, *p* < 0.001, *d*_z_ = 2.07. Personal situations were also perceived as more burdensome than global situations, *M*_Strain_personal_ = 78.4, *M*_strain_global_ = 55.9, *t*(211) = 13.45, *p* < 0.001, *d*_z_ = 0.92, and as having stronger negative effects on mental health, *M*_MentalHealth_personal_= 76.1, *M*_MentalHealth_global_ = 61.5, *t*(210) = 15.28, *p* < 0.001, *d*_z_ = 1.05.

The hierarchical multiple regression conducted to test the first part of the main hypothesis produced the following results (see Table 1): Model 1, including only situation type as a predictor, was already significant and explained 29.4% of the variance in the perceived impact on mental health, *F*_(1, 421)_ = 174.93, *p* < 0.001, Situation: *b* = −14.52, *t* = −13.23, *p* < 0.001. With personal situations coded as 1 and global situations coded as 2, this result indicates that personal challenges had a stronger negative impact on mental health than globally challenging situations. In Model 2, perceived control was added as a predictor. The model remained significant, *F*_(2, 420)_ = 87.26, *p* < 0.001, *R*^2^ = 0.294, but perceived control did not contribute significantly to the prediction, *b* = −0.00, *t* = −0.07, *p* = 0.948. In Model 3, perceived responsibility was added, increasing the explained variance in the perceived impact on mental health to 37.7%, *F*_(3, 419)_ = 84.37, *p* < 0.001. At this stage, situation, perceived control, and perceived responsibility all emerged as significant predictors: Situation: *b* = −8.10, *t* = −4.72, *p* < 0.001; Perceived control: *b* = −0.14, *t* = −4.17, *p* < 0.001; Perceived responsibility: *b* = 0.26, *t* = 7.47, *p* < 0.001. Perceived control and responsibility showed opposite effects, with higher perceived control being associated with less negative impact on mental health. In Model 4, strain was added as an additional predictor. The model remained significant, and the explained variance increased substantially to 53.8%, *F*_(4, 418)_ = 121.90, *p* < 0.001. Strain and situation were the strongest predictors, but perceived responsibility and perceived control also remained significant and continued to exert opposing effects: Situation: *b* = −6.94, *t* = −4.68, *p* < 0.001; Perceived control: *b* = −0.01, *t* = −3.30, *p* = 0.001; Perceived responsibility: *b* = 0.10, *t* = 2.90, *p* = 0.004; Strain: *b* = 0.31, *t* = 12.11, *p* < 0.001. Similar relations were also evident in the separate analyses—although they were more pronounced in the analysis of personal situations than in that of global situations (see Supplement).

**Table 1:** Hierarchical regression analyses predicting impact on mental health from situation (personal/global), control, responsibility, and strain.

Step	Model	*R* ^2^	Test	Predictor	Coefficent
Step 1	Mental health ~ situation	29.4%	*F*_(1, 421)_ = 174.93, *p* < 0.001	Situation	*b* = −14.52, *t* = −13.23, *p* < 0.001
Step 2	Mental health ~ situation + perceived control	29.4%	*F*_(2, 420)_ = 87.26, *p* < 0.001	Situation	*b* = −14.59, *t* = −9.25, *p* < 0.001
				Perceived control	*b* = −0.00, *t* = −0.07, *p* = 0.948
Step 3	Mental health ~ situation + perceived control + perceived responsibility	37.7%	*F*_(3, 419)_ = 84.37, *p* < 0.001	Situation	*b* = −8.10, *t* = −4.72, *p* < 0.001
				Perceived control	*b* = −0.14, *t* = −4.17, *p* < 0.001
				Perceived responsibility	*b* = 0.26, *t* = 7.47, *p* < 0.001
Step 4	Mental health ~ situation + perceived control + perceived responsibility + perceived strain	53.8%	*F*_(4, 418)_ = 121.90, *p* < 0.001	Situation	*b* = −6.94, *t* = −4.68, *p* < 0.001
				Perceived control	*b* = −0.10, *t* = −3.30, *p* = 0.001
				Perceived responsibility	*b* = 0.10, *t* = 2.90, *p* = 0.004
				Perceived strain	*b* = 0.31, *t* = 12.11, *p* < 0.001

Higher impact on mental health scores reflect a stronger negative effect on mental health (see Methods).

**Figure 4:**
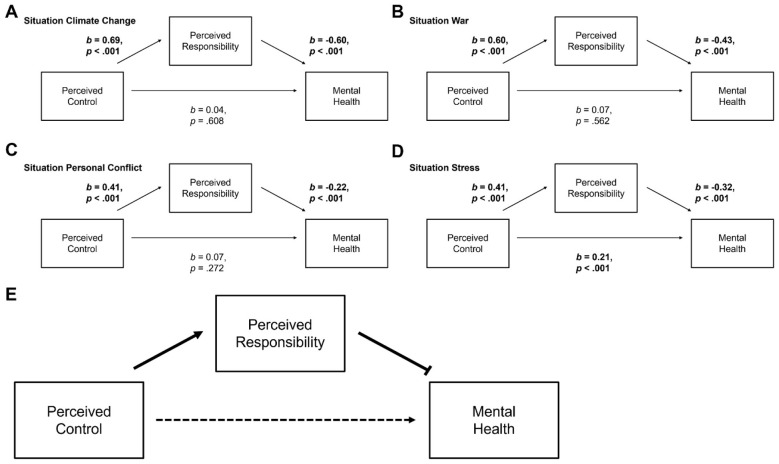
Mediation models: perceived control, responsibility and impact on mental health in challenging situations. **(A)** Situation: Climate Change. **(B)** Situation: War. **(C)** Situation: Personal. Conflict. **(D)** Situation: Stress. **(E)** Mediation model across all situations. The dashed line signifies a situation-specific effect. For these analyses, impact on mental health ratings were reverse-coded, i.e., higher scores reflect a more positive impact on mental health.

Results of the exploratory mediation analyses^5^ (see Figure 4) showed that perceived control was significantly associated with perceived responsibility across all situations (path a_climate_: *b* = 0.69, *p* < 0.001; path a_war_: *b* = 0.60, *p* < 0.001; path a_conflict_: *b* = 0.41, *p* < 0.001; path a_stress_: *b* = 0.41, *p* < 0.001) (see Figures 4A–D). In turn, perceived responsibility significantly predicted poorer mental health in all contexts (path b_climate_: *b* = −0.60, *p* < 0.001; path b_war_: *b* = −0.43, *p* < 0.001; path b_conflict_: *b* = −0.22, *p* < 0.001; path b_stress_: *b* = −0.32, *p* < 0.001).

The indirect path (a^*^b) was significant for all situations (a^*^b_climate:_ *b* = −0.41 95%CI [−0.54; −0.30], *p* < 0.001; a^*^b_war:_ *b* = −0.26 95%CI [−0.43; −0.13], *p* < 0.001; a^*^b_conflict:_ *b* = −0.09 95%CI [−0.17; −0.03], *p* < 0.001; a^*^b_stress:_ *b* = −0.13 95%CI [−0.20; −0.07], *p* < 0.001).

However, a direct effect of perceived control on mental health (path c) emerged only in the stress-situation, while no significant direct effects were observed in the other contexts (path c_climate:_ *b* = 0.04 95%CI [−0.12; 0.19], *p* = 0.608; path c_war:_ *b* = 0.07 95%CI [−0.15; 0.30], *p* = 0.562; path c_conflict:_ *b* = 0.07 95%CI [−0.05; 0.19], *p* = 0.272; path c_stress:_ *b* = 0.21 95%CI [0.13; 0.29], *p* < 0.001). Thus, for all situations, perceived control affected mental health negatively by strengthening perceived responsibility. Complete mediation models were evident for all situations except of the stress-situation, were perceived control additionally affected mental health positively via a direct path (Figure 4).^6^

The ANOVA conducted as a preliminary analysis of the emotional effects for the second part of the hypothesis revealed significant main effects for both the factor situation, *F*_(3, 600)_ = 38.24, *p* < 0.001, η_p_^2^ = 0.16, ε = 0.83 (GG-corrected) and the factor emotion, *F*_(7, 1400)_ = 240.27, *p* < 0.001, η_p_^2^ = 0.55, ε = 0.67 (GG-corrected). In addition, a statistically significant interaction effect between situation and emotion was found, *F*_(21, 4200)_ = 55.74, *p* < 0.001, η_p_^2^ = 0.22, ε = 0.74 (GG-corrected) (see Figure 3B).

Based on these findings indicating that the mean emotion ratings differed significantly, both across situations and across emotions, we opted to include all four situations in the subsequent analyses rather than average over situation type. For the sake of simplicity—and despite the significant differences between emotions found in the preliminary ANOVA—the different negative emotions were initially analyzed together.

The results of the multiple regression showed that Model 1, which included only situation as a predictor, was already significant and explained 5.6% of the variance in negative emotions, *F*_(1, 843)_ = 49.88, *p* < 0.001; *b* = 4.06, *t* = 7.06, *p* < 0.001 (see Table 2). Model 2, which additionally included perceived control as a predictor, was also significant, but still accounted for only a relatively small proportion of the variance in negative emotion ratings (6.6%), *F*_(2, 842)_ = 29.54, *p* < 0.001. Both situation and perceived control were significant predictors, Situation: *b* = −2.79, *t* = −3.88, *p* < 0.001; Perceived control: *b* = 0.08, *t* = 2.96, *p* = 0.003. Thus, perceived control was positively associated with higher negative emotions, contrary to our hypothesis. When perceived responsibility was added in Model 3, the model remained significant, and the explained variance increased to 24.9%, *F*_(3, 841)_ = 92.97, *p* < 0.001. Perceived responsibility emerged as the strongest significant predictor, while the effect of situation was no longer significant, perceived responsibility: *b* = 0.41, *t* = 14.34, *p* < 0.001; Situation: *b* = 0.91, *t* = 1.31, *p* = 0.190. Notably, after adding perceived responsibility, the impact of perceived control changed direction and now predicted lower levels of negative emotions, consistent with the hypothesis, *b* = −0.17, *t* = −5.80, *p* < 0.001. Model 4, which additionally included strain as a predictor, significantly explained a much larger proportion of the variance in negative emotions (52.4%), *F*_(4, 840)_ = 231.51, *p* < 0.001. Strain became the strongest predictor, while the effects of perceived responsibility and perceived control were attenuated, Strain: *b* = 0.48, *t* = 22.05, *p* < 0.001; Perceived responsibility: *b* = 0.22, *t* = 9.08, *p* < 0.001; Perceived control: *b* = −0.12, *t* = −5.14, *p* < 0.001. Perceived responsibility and perceived control retained their opposing directions of effect. In addition, situation again emerged as a significant predictor, *b* = 2.62, *t* = 4.70, *p* < 0.001.

**Table 2:** Hierarchical regression analyses predicting negative emotion from situation (conflict, stress, climate, war), control, responsibility, and strain.

Step	Model	*R* ^2^	Test	Predictor	Coefficent
Step 1	Negative emotion ~ situation	5.6%	*F*_(1, 843)_ = 49.88, *p* < 0.001	Situation	*b* = – 4.06, *t* = −7.06, *p* < 0.001
Step 2	Negative emotion ~ situation + perceived control	6.6%	*F*_(2, 842)_ = 29.54, *p* < 0.001	Situation	*b* = −2.79, *t* = −3.88, *p* < 0.001
				Perceived control	*b* = 0.08, *t* = 2.96, *p* = 0.003
Step 3	Negative emotion ~ situation + perceived control + perceived responsibility	24.9%	*F*_(3, 841)_ = 92.97, *p* < 0.001	Situation	*b* = 0.91, *t* = 1.31, *p* = 0.190
				Perceived control	*b* = −0.17, *t* = −5.80, *p* < 0.001
				Perceived responsibility	*b* = 0.41, *t* = 14.34, *p* < 0.001
Step 4	Negative emotion ~ situation + perceived control + perceived responsibility + perceived strain	52.4%	*F*_(4, 840)_ = 231.51, *p* < 0.001	Situation	*b* = 2.62, *t* = 4.69, *p* < 0.001
				Perceived control	*b* = −0.12, *t* = −5.14, *p* < 0.001
				Perceived responsibility	*b* = 0.22, *t* = 9.08, *p* < 0.001
				Perceived strain	*b* = 0.48, *t* = 22.05, *p* < 0.001

When emotions were considered separately, similar patterns could be observed-with the exception of anger. Guilt and shame showed the strongest influence of perceived responsibility and the most significant change in the role of perceived control. Significant effects were also observed for helplessness and fear. The relations were weaker for frustration and sadness, and no significant effect was found for anger. In an exploratory analysis of the positive emotion of hope, perceived control emerged as the only significant positive predictor (see Supplement for details).

### 3.3 Trait agency experience and use of problem-focused coping strategies (hypothesis 3)

#### 3.3.1 Analysis plan

The third hypothesis predicted that a more pronounced trait sense of agency would be associated with more frequent self-reported use of control-related coping strategies. To test this hypothesis, Pearson correlation analyses were conducted between SoAS scores and the reported frequency of use of various coping strategies.^7^

#### 3.3.2 Key findings

Consistent with the third hypothesis, the results revealed a small but significant positive correlation between SoAS scores and the reported frequency of using the control-related coping strategy “Focus on Taking Actions,” *r*(206) = 0.23 95%CI [0.06;0.37], *p* < 0.001. Likewise in line with the hypothesis, there was a small but significant negative correlation between SoAS scores and the use of the passive strategy “Giving Up Responsibility,” *r*(206) = −0.15 95%CI [−0.29; −0.01], *p* = 0.027. Interestingly, slightly higher correlations emerged between SoAS scores and the frequency of other coping strategies, in particular “Acceptance,” *r*(206) = 0.22 95%CI [0.07; 0.37], *p* = 0.002, and “Optimism,” *r*(206) = 0.24 95%CI [0.09; 0.38], *p* < 0.001. Additionally, a negative correlation was observed with the coping strategy “Professional Support,” *r*(206) = −0.19 95%CI [−0.34; −0.02], *p* = 0.007.

### 3.4 Helpfulness of control-related and other coping strategies (hypothesis 4)

#### 3.4.1 Analysis plan

**Figure 5:**
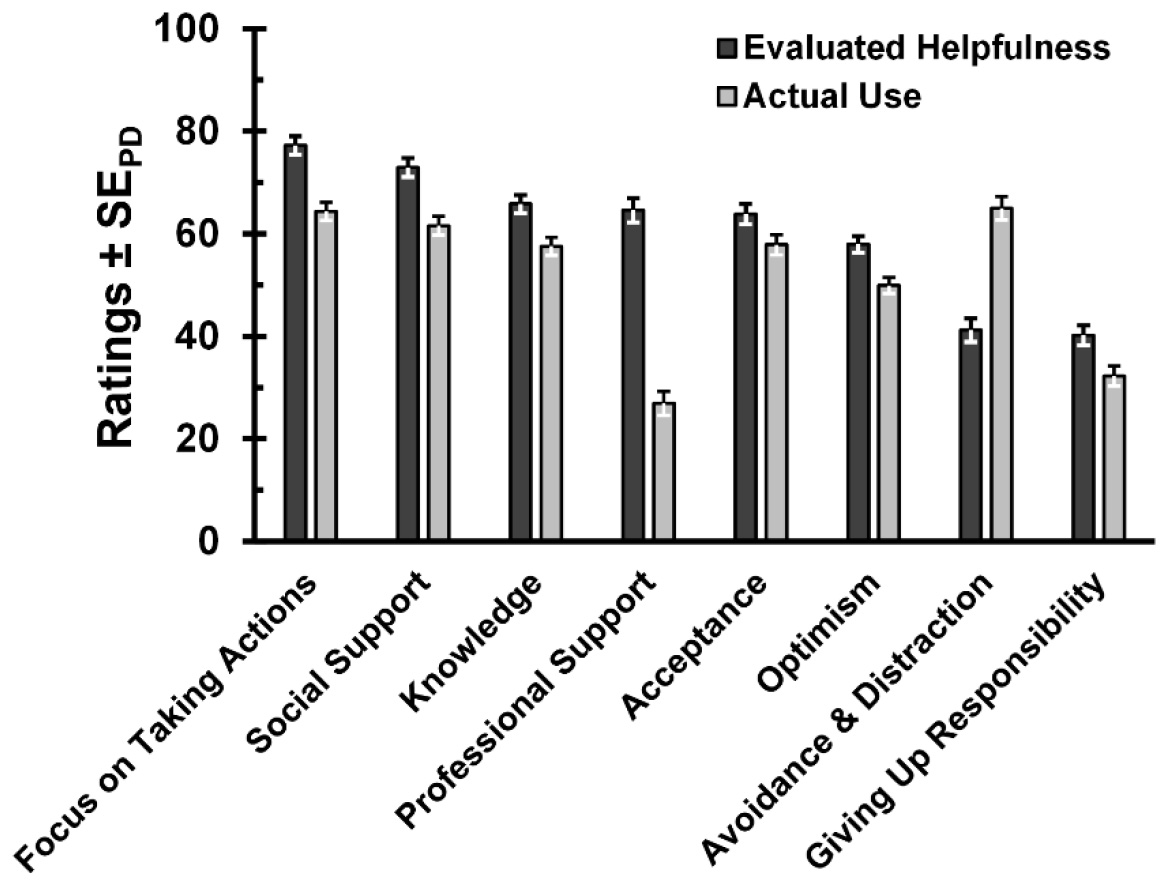
Perceived helpfulness and actual use of different coping strategies. Error bars indicated standard errors of paired differences (Pfister and Janczyk, 2013).

Hypothesis 4 proposed that control-related coping strategies would be perceived as more helpful than other strategies. To test this hypothesis, a repeated measures ANOVA was conducted. This analysis aimed at determining whether participants' evaluations differed significantly across the various coping strategies. As an exploratory extension, the frequency of use was included as a second factor. Consequently, a 2 (Evaluation Type: frequency of use vs. perceived helpfulness) × 8 (Coping Strategies) repeated measures ANOVA was performed to analyze the ratings across coping strategies (see Figure 5).

To further examine whether control-related strategies were perceived as more helpful than other strategies, multiple paired-sample *t*-tests were conducted. Specifically, the control-related strategy “Focus on Taking Actions” was compared to the less control-related strategies “Avoidance & Distraction,” “Acceptance,” “Optimism,” “Social support,” “Professional support,” “Giving Up Responsibility,” and “Knowledge” with regard to their perceived helpfulness.

In addition, exploratory analysis further examined the frequency of use and perceived helpfulness of the coping strategies. Follow-up paired sample *t*-tests were conducted to provide a more detailed understanding of the relationship between these two evaluation aspects.

#### 3.4.2 Key findings

Figure 5 shows the perceived helpfulness and actual use of different coping strategies. The analysis of main effects of the ANOVA revealed a significant effect of Evaluation Type, *F*_(1, 184)_ = 70.88, *p* < 0.001, η_p_^2^ = 0.28, as well as a significant main effect of Coping Strategy, *F*_(7, 1288)_ = 59.86, *p* < 0.001, η_p_^2^ = 0.25, ε = 0.82 (GG-corrected). These findings indicate significant differences both between evaluation types and across the eight coping strategies. Furthermore, the interaction between Evaluation Type and Coping Strategy was also significant, *F*_(7, 1288)_ = 63.70, *p* < 0.001, η_p_^2^ = 0.26, ε = 0.83 (GG-corrected).

The results of the paired-sample *t*-tests revealed significant differences in favor of the control-related strategy “Focus on Taking Actions” for all comparisons. The largest significant mean difference emerged between “Focus on Taking Actions” and “Avoidance & Distraction,” with *M*_*FocusonTakingActions*_= 77.2 and *M*_Avoidance&Distraction_ = 41.2, *n* = 212, *t*(211) = 13.72, *p* < 0.001, *d*_z_= 0.94. Accordingly, the active, control-related strategy “Focus on Taking Actions” was perceived as significantly more helpful than the passive strategy “Avoidance & Distraction.”

Significant differences with medium to large effect sizes in favor of “Focus on Taking Actions” were also observed in comparison to the strategies “Optimism” (*M*_*Focus on Taking Actions*_= 77.2^8^, *M*_*Optimism*_ = 57.9, *t*(211) = 10.04, *p* < 0.001, *d*_*z*_ = 0.69), “Acceptance” (*M*_*Focus on Taking Actions*_ = 77.2, *M*_*Acceptance*_ = 63.8, *t*(211) = 6.72, *p* < 0.001, *d*_*z*_ = 0.46), “Knowledge” (*M*_*FocusonTakingActions*_ = 77.4, *M*_*Knowledge*_ = 65.8, *t*(209) = 6.53, *p* < 0.001, *d*_*z*_ = 0.45), and “Professional Support” (*M*_*Focus on Taking Actions*_ = 78.1, *M*_*Professional Support*_ = 64.6, *t*(188) = 6.28, *p* < 0.001, *d*_*z*_ = 0.46). The smallest mean difference was observed between “Focus on Taking Actions” and “Social Support” (*M*_*Focus on Taking Actions*_= 77.2, *M*_*Social Support*_ = 72.9; *t*(209) = 2.53, *p* =0.012, *d*_*z*_ = 0.17). Interestingly, the comparison of actual use and helpfulness revealed that most coping strategies were used considerably less than they were perceived as helpful, including active coping (*M*_use_ = 64.4, *M*_helpfulness_ = 77.6, *t*(210) = −7.38, *p* < 0.001, *d*_z_ = −0.51; for more details, see Supplement). The sole exception to this rule was the *Avoidance and Distraction* strategy which was used far more than it was considered helpful, indicating potentially unhealthy real-life coping habits (*M*_use_ = 65.0, *M*_helpfulness_ = 41.2, *t*(211) = 10.32, *p* < 0.001, *d*_z_ = 0.71).

### 3.5 Exploratory analyses

In addition to the hypotheses outlined above, additional exploratory analyses were conducted, focusing, among other things, on the overlap between coping strategies perceived as helpful and those actually used. Differences in responses to globally vs. personally challenging situations were also examined. Furthermore, some analyses were conducted using the General Self-Efficacy Scale instead of trait SoA. Due to the limited scope of this manuscript, not all analyses can be discussed in detail and are therefore reported in the Supplement.

## 4 Discussion

The aim of this study was to examine the relationship between agency experience and mental health, both in specific situations (state level) and as a trait. Additionally, the study investigated the use and perceived helpfulness of various coping strategies differing in their association with control experience.

### 4.1 Interpretation of findings and practical implications

#### 4.1.1 Trait agency experience and mental health

The assumption that higher agency experience is associated with better mental health and fewer self-reported psychological problems was largely confirmed by the results. The observed positive correlation between general mental health ratings and the mean score on the Sense of Agency Scale indicates that a higher trait-level agency experience is indeed associated with better mental health. Higher trait agency experience was also associated with the absence of clinically relevant mental disorders. Considering that depression and anxiety disorders were the most common conditions among participants, this finding aligns with previous research and theoretical models linking reduced perceived control to depression and anxiety (e.g., Chorpita and Barlow, 1998; Gallagher et al., 2014; Liu et al., 2015; Ly et al., 2019). Notably, significant effects were observed only in relation to clinically relevant mental disorders, but not for subclinical mental health problems. These findings may suggest that the hypothesized relationship between agency experience and mental health becomes particularly evident in cases of pronounced, clinically relevant impairments. Nevertheless, the general correlation between mental health and the Sense of Agency Scale indicates that a fundamental association may exist across all levels of mental health - albeit with varying degrees of strength.

It thus appears reasonable to consider the agency experience as a potential target for interventions aimed at improving mental health (see also Ly et al., 2019). As previous studies have also demonstrated (Adler, 2011; Gallagher et al., 2014), enhancing agency experience may be associated with improved mental health and reduced psychopathology, particularly with regard to anxiety disorders and depression-both of which were also the predominant psychological conditions observed in our study.

#### 4.1.2 Perceived control and mental health in challenging situations

In our initial hypotheses, we assumed a stronger sense of control to be positively related to mental health, also in specific challenging situations. However, this simple relationship was not confirmed; instead, a more complex picture formed in our data: Indeed, perceived control did not seem to have a significant effect on mental health on its own; if anything, it seemed to have a descriptively, detrimental effect. However, when responsibility was added as a predictor in our models, the picture changed fundamentally: Perceived responsibility emerged as a significant predictor of stronger negative effects on mental health, while the role of perceived control shifted to predicting better mental health. For personal situations, the association between higher perceived control and better mental health became stronger. For global situations, the adverse association between perceived control and mental health outcomes disappeared. Taken together, greater perceived control was associated with fewer negative effects on mental health –in line with our hypothesis– but only if perceived responsibility was included in the model hinting at both, shared and independent, behavioral variance and a potential double role of perceived control in the context of mental health. Indeed, mediation analyses revealed that perceived control was associated with mental health in all situations mostly indirectly via perceived responsibility, and thus in a negative fashion.^9^ In situations of personal stress, however, perceived control also affected mental health positively via a direct path. These analyses indicate that perceived control may be a surprisingly double-edged sword for mental health.

Perceived control and experienced responsibility are conceptually closely intertwined (Schwarz, under review). This suggests that they would operate in a similar direction, with responsibility initially conceptualized as an additional measure of agency experience—consistent with approaches taken in other studies (Bart et al., 2023). As a downstream consequence of authorship attribution, the inclusion of perceived responsibility allowed us to reflect both subcomponents of agency experience (i.e., perceived control and authorship attribution) in the participants' reports. Our assumption that both would affect mental health in a similar fashion, however, was not reflected in our data. This is in line with findings that suggest that authorship experience and perceived control may rely on different mechanisms to emerge: Whereas perceived control seems to rely heavily on a comparison of the a priori goal and the achieved outcome (e.g., Schreiner et al., 2025), sometimes even irrespective of actual control (e.g., Ueda et al., 2021), this explanation for the emergence of experienced agency only holds true as long as authorship assumptions can be upheld (e.g., Ueda et al., 2021). Authorship assumptions therefore seem to represent a prerequisite for the emergence of agency experience. Thus, perceived control and perceived responsibility, while sharing certain basic assumptions, seem to incorporate different emphases of the agency experience that may affect mental health differently depending on the situation.

The analyses of negative emotions further support this interpretation. While perceived control initially appeared to be associated with greater negative affect, this effect reversed once responsibility was included, suggesting that perceived responsibility may obscure the beneficial role of perceived control. Across specific emotions, this masking effect was particularly evident for guilt and shame-emotions that are closely tied to the experience of responsibility. They revealed the strongest impact of perceived responsibility and the clearest shift in the role of perceived control. Stronger, though somewhat smaller, effects also emerged for helplessness and anxiety, both of which have been repeatedly tied to perceptions of control in prior research (e.g., Chorpita and Barlow, 1998; Miller and Seligman, 1975). For frustration and sadness, the associations were weaker, and no significant link was found for anger. An exploratory finding of particular interest was the association between perceived control and the positive emotion of hope. Perceived control was positively related to hope, pointing to its potential role in fostering adaptive positive affect. Perceived responsibility, however, did not contribute to this relationship.

Overall, these findings highlight the dual role of perceived control, with its positive effects becoming visible only once its contributing aspects to perceived responsibility are accounted for.

This somewhat unexpected finding in the context of mental health is in line with previous evidence that showcases that exerting more control-and thus taking on more responsibility-especially in social situations may be stressful and leading to more dissatisfaction with outcomes (Schwarz et al., 2023). From a practical perspective, caution is warranted when aiming to enhance perceived control in stressful contexts to improve mental health. Ideally, interventions would increase perceived control without simultaneously raising the sense of responsibility-a challenging goal, given the strong conceptual link between the two constructs (Haggard and Tsakiris, 2009; Moore, 2016). Nevertheless, the differing association between subcomponents of agency experience, i.e., perceived control and authorship assumption, may prove fruitful here. Situations, in which control is high, but authorship diminished, may provide an optimal ground for effective strategies improving mental health. A simple situation may involve social factors: As soon as more than one person is involved authorship assumptions are divided (Beyer et al., 2017; Darley and Latané, 1968; Schwarz et al., 2023), even when control remains relatively high.

Interestingly, this dual effect of control emerged less strongly in global situations than in personal scenarios. Indeed, our analyses revealed that both perceived control and responsibility were strongly dependent on the type of situation. In personal scenarios (stress and conflict), levels of perceived control and responsibility were substantially higher than in global scenarios (war and climate change). These findings are consistent with previous research that also demonstrated stronger experiences of control in personal compared to global contexts (McElwee and Brittain, 2009; Wenglert and Rosén, 2000). Supporting this interpretation, our study showed lower variance explained by perceived control, perceived responsibility, and strain in global compared to personal challenging situations. This suggests that while global issues are not irrelevant to mental health, the greater and more immediate threat stems from everyday stressors such as interpersonal conflicts and daily pressures. Consequently, personal situations may offer greater potential as targets for mental health interventions because they are associated with higher perceived control and perceived responsibility, and thus, potentially, with greater opportunities for influence and change.

#### 4.1.3 Trait agency experience and use of control-related coping

The correlations between the Sense of Agency Scale and the use of various coping strategies revealed a positive association between SoAS scores and the use of the control-related strategy “Focus on Taking Actions,” as well as a corresponding negative association with the control-distant strategy “Giving Up Responsibility,” These findings support the hypothesis that a higher trait agency experience is associated with more frequent use of control-related coping strategies. However, it should be noted that the relatively small effect sizes point to limited practical significance. Interestingly, the strongest correlations were found not with the expected strategies, but rather with “Acceptance” and “Optimism.” Thus, a pronounced trait agency experience appears to be associated not only with active, control-related coping but also with a positive and accepting approach to challenging situations. A speculative interpretation may be that stronger agency assumptions simply allow are more positive outlook, making strategies such as optimism or even acceptance more viable. Indeed, previous evidence indicates that agency experience is a generally rewarding phenomenon that individuals seek out deliberately (Reis et al., 2023; Schwarz, 2025) if they are not hampered situationally with increased pressures such as heightened social responsibility (Schwarz et al., 2023). Thus, a general strong assumption of agency may have positive effects even through non-directly control-related coping strategies. This interpretation is also in line with the positive association of trait agency experience and general mental wellbeing.

#### 4.1.4 Superiority of control-related coping strategies

The final hypothesis, positing that individuals perceive control-related coping strategies as generally more helpful than other types of strategies, was supported by the results of our analysis. Results from *t*-tests conducted following significant main effects in the ANOVA showed that the strategy most strongly associated with perceived control–“Focus on Taking Actions”- was indeed rated as significantly more helpful than all other listed coping strategies. The strategy “Social Support” was also rated as highly helpful (second place), although it is difficult to categorize clearly in terms of conveyed control. The next most helpful strategies, “Knowledge” and “Professional Support,” allow for a somewhat stronger link to perceived control than the strategies rated as least helpful: “Acceptance,” “Optimism,” and finally, “Avoidance & Distraction.” Thus, consistent with previous findings by Dijkstra and Homan (2016) and Litman and Lunsford (2009), participants in our study perceived active, problem-focused coping strategies as most helpful (see Figure 5).

Moreover, exploratory analyses revealed that while the actual use of control-related coping strategies was not strongly associated with general agency experience, the perceived helpfulness of control-related coping strategies did show a moderate association with trait SoA. For example, the correlation between the SoAS and the perceived helpfulness of “Focus on Taking Actions” (*r*(184) =0.39 95%CI [0.23; 0.54], *p* < 0.001) was notably higher than the correlation with its actual use evaluation (*r*(206) = 0.23 95%CI [0.07; 0.37], *p* < 0.001). Taken together, our results indicate associations between the use and perceived helpfulness of control-related coping strategies with trait SoA. However, this association is stronger for the perceived helpfulness than the actual use, hinting that an individual's behavior may not always mirror their assumptions.

This interpretation is also in line with the exploratory comparison of use and perceived helpfulness of coping strategies: Comparisons of frequency of use with perceived helpfulness revealed significant differences across all strategies. The strategy “Avoidance & Distraction” was particularly striking: although it was rated as the second least helpful, it was the second most frequently used (after “Focus on Taking Actions”). For all other strategies, the effects were reversed—helpfulness ratings were significantly higher than usage ratings. Even “Focus on Taking Actions”—which is of particular interest in the context of this study due to its relation to perceived control—was used less frequently than would be expected given its high helpfulness rating. Interestingly, the discrepancy between actual use and perceived helpfulness of coping strategies was reduced for higher trait SoA (*r*(210) = −0.20 95%CI [−0.34; −0.05], *p* = 0.004).

Thus, there seems to be already a solid understanding of the value of control-oriented coping strategies, but this knowledge is often not translated into practice. Addressing these discrepancies, especially in those strategies considered as most helpful (e.g. “Focus on Taking Actions” and “Social Support”), could offer a potential avenue for improving coping behavior and, consequently, mental health. To fully harness the positive effects of these strategies - effects that were both assumed by participants and supported by previous studies (e.g., Chao, 2011; Dijkstra and Homan, 2016; Koeske et al., 1993)-it appears beneficial to promote their use in interventions aimed at managing challenging situations and improving mental health. Further potential lies in the passive strategy of “Avoidance & Distraction,” which, despite being perceived as relatively unhelpful, is used frequently. Another notable discrepancy emerged in the strategy of “Professional Support”: although it was rated as highly helpful, it was used the least among the offered coping strategies. Improving access to and reducing stigma around professional mental health services across different contexts could play a key role here—ideally framed as empowering support for self-help, ultimately fostering more sustained active coping.

#### 4.1.5 Potential practical implications

The present study aimed to elucidate the associations of experienced agency and mental health in a broad cross-situational evaluation, as well as in specific challenging situations. Our data reveal that while agency experience and mental health positively correlated generally, in specific situations, increasing agency perception may not necessarily improve mental health; instead, we found that while perceived control may positively impact mental health directly, it also may have a more pronounced negative impact via its association with perceived responsibility. As this is only the first study to look into and find this dissociation, implications have to be taken cautiously. However, while unexpected, the found pattern was profoundly systematic, re-appearing in all relevant analyses for our specific situations aside from the only tested positive emotion, i.e., hope, and anger. If this pattern holds true also in future studies, interventions might have to take care to emphasize control rather than responsibility for patients, e.g., via distributing perceived responsibility by focusing on social support or further social variables. Alternatively, the reported negative impact of perceived responsibility on mental health might be especially strong in the present study because our participants could not alleviate stressful situations by their own means. Potentially, giving participants or patients the tools to utilize their perceived responsibility by becoming active in changing the stressful situations might alleviate the negative impact on mental health we found. For mental distress based on globally challenging situations, however, this might be more difficult to achieve than for personally challenging situations. Future studies thus have to detail if such changes in study design might result in different result patterns.

### 4.2 Limitations

Participants of this study were predominantly young adults with relatively high educational background (see Supplement). Therefore, the generalizability of findings to middle-aged or older populations may be limited, as age-related differences in agency perceptions, coping strategies and mental health cannot be ruled out. In addition, as the study was conducted in a German-speaking context, cultural factors may have shaped the observed relationships, and future research should examine whether the results replicate in other cultural settings.

Moreover, as this study employed a correlational rather than an experimental approach, no causal inferences can be drawn based solely on the present study. Instead, it aims to inspire targeted experimental approaches that take into account the found associations between perceived control, perceived responsibility, mental health, and coping strategies. Since our approach is correlational and not experimental regarding the measured subcomponents of agency experience and mental health, our data do not specify the direction of the association, and do not preclude the influence of further variables not measured in this study. Nevertheless, the usage of the chosen variables was based on unidirectional conceptual a-priori assumptions, i.e., that that perceived control and responsibility may impact mental strain, and mental strain may be closely related to or impact mental health.

As all data were based on self-reports, potential biases such as response tendencies, social desirability effects, limited self-awareness, or misinterpretation of survey items cannot be precluded. Nevertheless, because explicit measures of agency experience are currently the least controversial approach (see e.g., Gutzeit et al., 2023; Hoerl et al., 2020; Schwarz and Weller, 2023; Tonn et al., 2021) and because these explicit measures are so well adaptable to diverse context and situations while retaining comparability, these self-reports still represent the best choice in our view.

Please note also that some constructs in this study were measured using only a single item (e.g., mental health), which may reduce the reliability and validity of those findings. Although single-item self-rated mental health measures are widely used in survey research, future studies could benefit from employing multi-item instruments to enable more nuanced assessments. Moreover, the analysis of specific stressful situations was based on only four self-selected scenarios. While data on participants' general perceptions of stressors (see Supplement) suggest a degree of representativeness, it remains possible that other types of situations would have yielded different results.

Finally, in this study only stressful contexts were examined. In positive contexts, the associations may differ: Greater perceived control and the accompanying sense of responsibility could enhance self-efficacy and experiences of success, thereby exerting stronger beneficial effects on mental health. This interpretation aligns with research linking optimism to perceived control over personal, potentially positive future events (e.g., McElwee and Brittain, 2009; Weinstein, 1980).

## 5 Conclusion

The present study portrays the associations of agency experience, mental health, and coping in two surprisingly contradictory ways. While mental health was generally positively associated with trait agency experience, for specific challenging situations, we found perceived control to hold a dual role: while it may increase mental health on its own, it may also diminish mental health via its direct influence on perceived responsibility. Moreover, whereas coping strategies with a stronger control component were perceived as particularly helpful, that perception was not necessarily reflected in actual use-a discrepancy that was again associated with trait sense of agency, indicating that behavior matches helpfulness assumptions more clearly when agency experience is strong.

Thus, interventions aimed at strengthening the agency experience therefore represent a promising avenue for improving mental health, though future research should focus on developing approaches that take the above findings into account and minimize potential negative effects of increased perceived responsibility.

## Data Availability

The datasets presented in this study can be found in online repositories. The names of the repository/repositories and accession number(s) can be found below: osf.io/e8r5h/.

## Footnotes

1 While self-efficacy is closely related concept, please note that it is generally perceived to be less action-specific and more abstract, and thus represents a more generalized, positive belief in personal competence and ability to engage in desired behavior successfully, rather than concrete control and authorship assumptions regarding specific action-effect episodes (e.g., Tapal et al., 2017).

2 Please note that experienced agency is a phenomenon most strongly influenced by situational variables, whereas individual aspects only seem to play a role in specific contexts (see, e.g., Schwarz et al., 2022). However, trait concepts of experienced agency (such as by Tapal et al., 2017) may still be informative regarding individual behavior or perception, especially when analyzed in the context of general, overarching inquiries rather than evaluations regarding specific situations (see, e.g., Schwarz et al., 2025).

3 Please note that this scale (very positive to very negative) was based on a pilot study in which participants evaluated this scale to be much easier and less error-prone in this context, i.e., after indicating their degree of mental strain with a high rating indicating high mental strain, than the perhaps more common scale ranging from very negative to very positive.

4 Please note that the self-efficacy scale correlated even more strongly with overall mental health (*r* =.50, *p* < .001). However, as our further focus of the present study was on experienced agency, also in the context of active coping strategies, and controllability and responsibility ratings in specific situations which are conceptually closer to experienced agency, we have opted to focus on SoAS scores throughout the main manuscript. Please find results for self-efficacy in the Supplement.

5 For the mediation analyses, “impact on mental health” was reverse-coded, so that higher scores reflect a more positive impact on mental health.

6 Please note that the present study is purely correlational. Causal relationships cannot be established with certainty; any causal inferences are not based on experimental procedures, but instead based on cautious conceptual analysis. That is, we based our survey on the conceptual assumption that perceived control and responsibility may impact mental strain, and mental strain may be closely related to or impact mental health. Based on previous literature and a-priori assumptions, we did not conceptualize that mental health would impact perceived responsibility in turn. Nevertheless, due to their correlational nature, our data do not preclude this possibility.

7 We used a single compound score for the SoAS, whereas the version of this scale comes with two subscales: Sense of Positive Agency and Sense of Negative Agency. These subscales emerged from a factor analysis of the validation data (Tapal et al., 2017). A similar two-factor structure was not equally apparent in the German translation (Bart et al., 2023), however, and the present data suggested either a single-factor or a three-factor solution instead.

8 Please note that depending on the comparison, means may change because some rating data for coping strategies were missing: n(Knowledge) = 210, n(Professional support) = 189, n(Social support) = 210, all other coping strategies n = 212.

9 An intriguing question for future research is whether perceived responsibility is indeed a mediating variable or whether it should be more seen as a moderator that determines when the impact of control on mental health is positive and when it is negative.
