# Supplementary material for: Mental health in challenging situations: how experienced agency affects coping and mental distress
Source: Front Psychol. 2026 May 13;17:1786909. doi: 10.3389/fpsyg.2026.1786909 (PMC13212201; doi:10.3389/fpsyg.2026.1786909)
Supplement: Supplementary file 1 [file Data_Sheet_1.pdf]

# Mental health in challenging situations: how experienced agency affects coping and mental distress

## Supplement

Luana Benz[1], Roland Pfister[2,3], & Katharina A. Schwarz*[2,3,4]

[1]Department of Psychology III, University of Würzburg, Germany
[2]Institute of Psychology, Trier University, Germany
[3]Institute for Cognitive and Affective Neuroscience (ICAN), Trier University, Germany
[4]Department of Behavioural and Cognitive Sciences, University of Luxembourg, Luxembourg

*Corresponding Author

# Table of Contents

*Sociodemographic Data* ........ *3*
Figure S1. Highest formal education. ........ 3
Figure S2. Household income per month. ........ 4
Figure S3. Living environment. ........ 5
*Questionnaire* ........ *6*
Mental health assessment ........ 6
Questions about specific stressful situations ........ 8
General questions about stressful situations ........ 9
Coping strategies ........ 10
Sense of Agency Scale ........ 12
Self-Efficiacy Scale ........ 12
*Supplementary Analyses* ........ *13*
Supplementary analyses related to Hypothesis 1 ........ 13
Figure S4. Mean General Self Efficacy Scale scores across different mental health status groups ........ 13
Supplementary analyses related to Hypothesis 2 ........ 14
Supplementary analyses related to Hypothesis 3 ........ 23
Supplementary analyses related to Hypothesis 4 ........ 24
Analysis of open questions regarding mental health ........ 25

# Sociodemographic Data

N = 212, mean age = 27.0 years, SE = 8.4, range: 19-68

**Gender**: 134 female, 75 male, 1 non-binary

### Highest formal education (completed)

Doctorate/ PhD: 2, Diploma, Magister, Master: 27, Bachelor´s degree: 36, Vocational training completed: 13, University entrance qualification (Abitur): 127, Intermediate school certificate: 5, Basic school certificate: 1, No school certificate: 0, Other certificate: 0, not specified: 1 (see Figure S1).

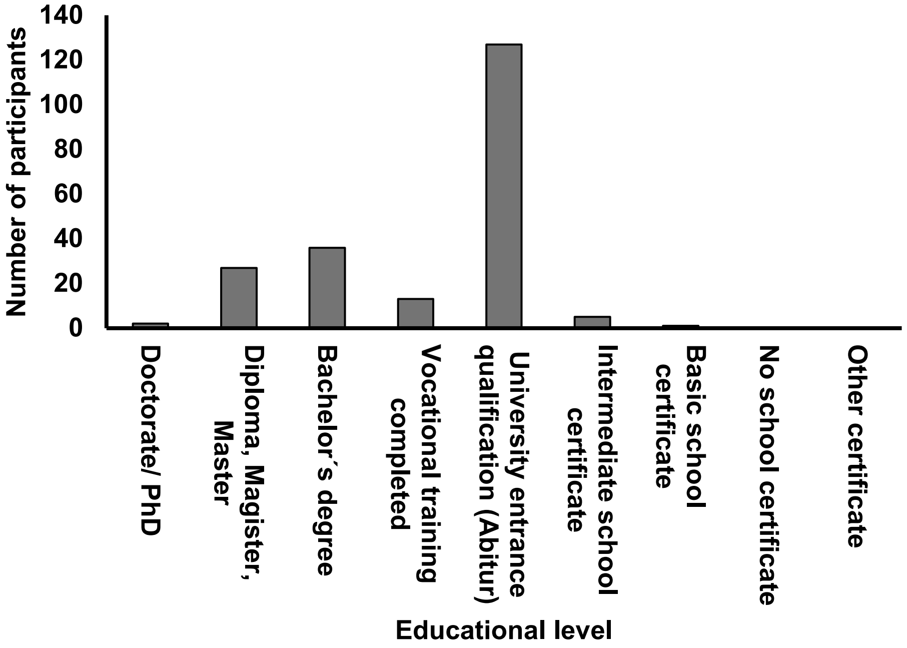


Figure S1. Highest formal education.

**Total household income per month:**

< 900€: 69, 900-1,500€: 36, 1,500-2,600€: 42, 2,600-3,600€: 18; 3,600-5,000€: 21; 5,000-10,000€: 9, >10,000€: 1, not specified: 16 (see Figure S2).

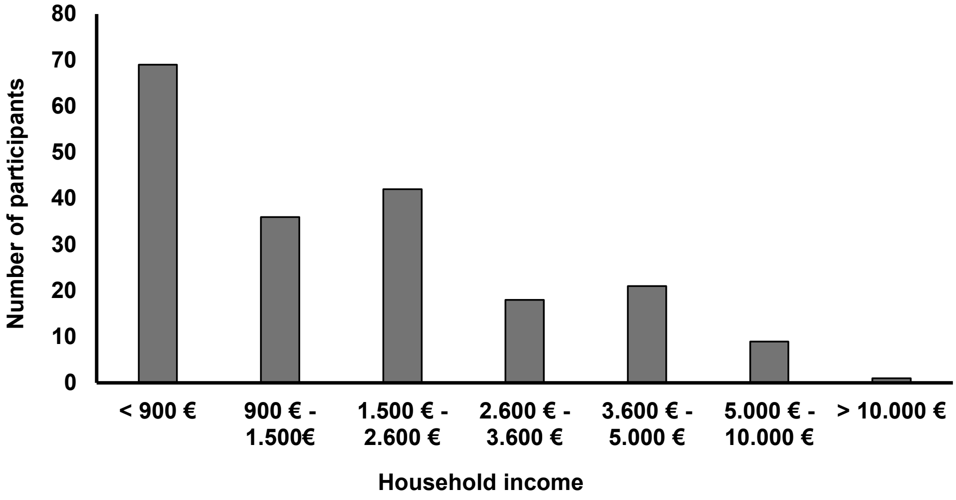


Figure S2. Household income per month.

**Adult household members**

1 adult: 99, 2 adults: 66, 3 adults: 26, 4 adults: 10, 5 adults:3, 6 adults: 0, 7 adults: 1, not specified: 7

**Children**
0 children: 24, 1 child: 7. 2 children: 13, 3 children: 2, not specified: 166

**Living country**

Germany: 210, Austria: 0, Switzerland: 0, other country: 2

**Living environment**

Rural area (population < 5,000, > 10km to the next town/city): 24, small town (population < 20,000, > 10km to the next town/city): 14, suburban area (population < 20000, < 10km to the next town/city): 13, medium-sized town (population between 20,000-200,000): 131, Large city (population > 200,000): 30 (see Figure S3).

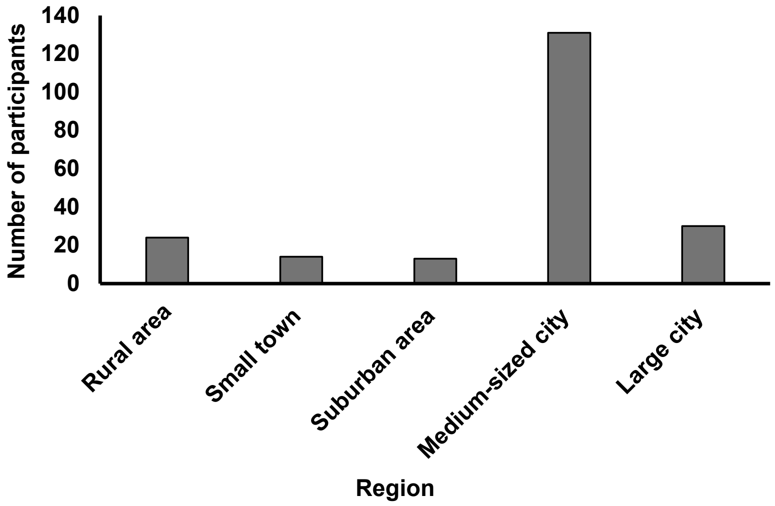


Figure S3. Living environment.

# Questionnaire

English translation

## Mental health assessment

Now we would like to ask you a few questions about your experiences with mental health.

To ensure that we are all on the same page, we would first like to briefly explain the term “mental health.” Please read the following definition carefully: *Mental health is not merely the absence of mental disorders. Mental health describes a state of well-being in which a person recognizes their own abilities, can cope with the normal stresses of life, can work productively, and is able to contribute to the community*. (World Health Organization)

* indicates a filter question that depending on the answer would open up different subsequent questions.

Have you already dealt with the topic of ‘mental health‘? (filter question) Options: Yes; No; No answer

***In what context have you already come into contact with the topic of mental health?** (Multiple selections possible)

Options: Own mental health; Mental health of social environment; General interest; other: [open text field]; no answer

**To what extent was mental health a relevant topic in your social environment?** (Multiple selections possible)

Options: Mental health problems in close social circles (e.g., partner, family, close friends, etc.); Use of support services (e.g., psychiatrist, psychotherapy) in close social circles; Mental health problems in the wider social environment (e.g., coworkers, acquaintances, etc.); Use of support services (e.g., psychiatrist, psychotherapy) in the wider social environment; Other: [open text field]; No answer

***To what extent have you dealt with your own mental health?** (Multiple selections possible)

Options: Interest or personal development; Subclinical, undiagnosed mental health issues (e.g., stress, temporary depressive mood, etc.); Diagnosed mental disorders (e.g., depression); Other: [open text field]; No answer

**You have indicated that you have had experience with subclinical, undiagnosed mental health problems. What specific problems did you have?** Please briefly describe them in keywords (e.g., stress, depressive mood, etc.): [open text field]; no answer

**You have indicated that you have had experience with diagnosed mental disorders. What specific mental disorders did you have?** Please briefly describe them in keywords (e.g., depression, obsessive-compulsive disorder, etc.): [open text field]; no answer

***Have you already sought professional help (e.g., family doctor, psychotherapy, medication) due to mental health issues?**

Options: Yes; No; No answer

**You have stated that you have already sought professional help for your mental health. What specific help did you receive?** (e.g., family doctor, psychotherapy, hospital stay, medication, etc.): [open text field]; No answer

**How would you rate your current mental health?** Remember: *Mental health is not just the absence of mental disorders. Mental health describes a state of well-being in which a person*

*recognizes their own abilities, can cope with normal stresses in life, can work productively, and is able to contribute to the community.* (World Health Organization)"
Slider scale: "very poor" to "very good"

## Questions about specific stressful situations

Now we will briefly describe specific situations to you and then ask you questions about them. Please answer honestly and to the best of your knowledge.

(The same questions, listed in the following section, were always asked about the following situations)

**Situation 1**: Imagine you have an important work/school project or task that you need to complete within a specified time frame. When you look at your calendar, you realize that this seems almost impossible to achieve.

**Situation 2:** You repeatedly have heated arguments with someone close to you (e.g., a family member or partner). Despite multiple attempts to find a solution, the conflict seems to escalate again and again.

**Situation 3:** Think about the increasing effects of climate change. In recent years, natural disasters such as floods, storms, and forest fires have become more frequent, causing damage and loss worldwide.

**Situation 4**: Armed conflicts and wars are taking place in various parts of the world, causing enormous human and social losses. Millions of people are forced to leave their homes and flee to safe areas, as well as cope with great losses and poverty.

**Questions asked about each of the situations:**

“What emotions does this situation trigger in you? Please rate how strongly the following emotions occur on a scale from ”not at all“ to ”very strongly“ as a result of the situation described above.”

Emotions: Anger/rage, fear, helplessness, guilt, shame, frustration, sadness, hope, other (specify): [open text field]

Slider scale for each emotion: “Not at all” to “Very strongly”; No answer

Please rate the situation described above in terms of the following aspects:

- ➢ How strongly do you feel you can control/influence the situation?
- ➢ How strongly can other people control/influence the situation?
- ➢ How responsible do you feel for this event?
- ➢ How much strain do you experience this situation?
- ➢ Do you feel that this situation is affecting your mental health?

Sider Scale for each emotion: “Not at all” to “Very strongly”; No answer

## General questions about stressful situations

The following questions are about your general perception of stressful events, detached from specific situations as much as possible. Please answer these questions honestly and to the best of your knowledge.

(The following questions were asked separately for personal and global situations:)

What **emotions** do you generally associate with the thought of personal (/ global) challenges and crises (e.g., interpersonal conflicts, illness, financial crises, etc.)? Please check the appropriate box (multiple selections possible).
→ Options: Anger/rage; fear; helplessness; guilt; shame; frustration; sadness; hope; other (please specify); no answer

How often do **thoughts and feelings** about personal (/global) challenges arise in you?
Slider scale: “Very rarely” to “Several times a day”; No answer

How often do you feel **powerless or overwhelmed** due to personal challenges or crises (e.g., financial difficulties, disputes, etc.) (/global challenges or crises, e.g., climate change, geopolitical tensions, etc.)?”
Slider scale: “Very rarely” to “Several times a day”; No answer

How strongly do you feel you can **control/influence** personal (/global) events?
Slider scale: “Not at all” to “Very strongly”; No answer

How strongly do you feel that **other people can control/influence** your personal (/global) events?
Slider scale: “Not at all” to “Very strongly”; No answer

How much **strain** do you experience when you are confronted with such stressful situations in your personal life (/ when you are confronted with such global challenges)?
Slider scale: “Not at all” to “Very strongly”; No answer

Do you feel that these personal (/global) events are affecting your **mental health**?
Slider scale: “Strong positive effects” to “Strong negative effects”; no answer

## Coping strategies

Different people deal with stressful crises and challenges in different ways. In this context, we talk about different *coping strategies*. Coping strategies describe attempts to cope with demands that arise from stressful events and overwhelm a person's resources (Lazarus & Folkman, 1984).

We would like to take a closer look at some coping strategies below. Please read the following explanations carefully:

- **Avoidance & Distraction:** I avoid dealing with the issue and try to distract myself (e.g., through hobbies, work, sports).
- **Acceptance**: I try to accept the situation as it is and adjust my focus and expectations accordingly.
- **Optimism**: I try to stay optimistic and think positively.
- **Social Support**: I seek social and emotional support and talk to friends or family about it, for example.
- **Professional Support**: I seek professional support (e.g., specialists, therapists, etc.).
- **Giving Up Responsibility**: I delegate the decision or task to competent others whose skills I trust.
- **Knowledge**: I am trying to learn more about the problem and gain a more fundamental understanding.
- **Focus on Taking Action**: I focus on what I can influence, take active action, and try to find solutions.

**Rating for frequency of use and helpfulness of coping strategies**

**Frequency of use**

Now think of a challenge or crisis that you found particularly stressful. What coping strategies did you use to deal with it? Please indicate **how often** you actually **use** the coping strategies listed below.

Slider scale: “Never” to “Almost always”; no answer
(Participants evaluated all the coping strategies mentioned above in randomized order)

**Helpfulness**

Think back to a challenge or crisis that you found particularly stressful. Which of the strategies do you find most helpful in dealing with such a challenge or crisis? Please indicate **how helpful** you consider the coping strategies below to be - as independently as possible from your own use of them.

Slider Scale: “Not helpful at all” to “Very helpful”; no answer
(Participants evaluated all the coping strategies mentioned above in randomized order)

**Ranking for frequency of use and helpfulness of coping strategies**

**Frequency of use**

Think back to a challenge or crisis that you found particularly stressful. What coping strategies did you use to deal with it? Please rank the coping strategies according to how often you actually use them. To do this, either double-click on the respective coping strategy or drag it to the desired position with the mouse (Rank 1 = most frequently, rank 8 = least frequently).

**Helpfulness**

Think back to a challenge or crisis that you found particularly stressful. Which of the strategies do you find most helpful in dealing with such a challenge or crisis? Please now rank the coping strategies according to how helpful you think they are – as independently as possible from your own use of them. To do this, either double-click on the respective coping strategy or drag it to the desired position with the mouse (Rank 1 = most helpful, rank 8 = least helpful).

(The coping strategies were listed again under the rankings and ratings: As a reminder, the strategies are shown again here with a brief explanation: [Explanations of the coping strategies])

**Open-ended questions about Coping**

Are there any other strategies that you often use or find particularly useful that have not been mentioned yet? Please list them briefly.

Other frequently used strategies: [open text field];
Other particularly helpful strategies: [open text field]

## Sense of Agency Scale

In the following, we would like to ask you a few questions about your perception of control. To do this, we will use the German version of an existing questionnaire (Sense of Agency Scale according to Bart et al., 2023). Please answer the following questions honestly and to the best of your knowledge.

Please indicate how much you agree with the following statements.

Items (presented in randomized order):

- Nothing I do is actually voluntary.
- The outcomes of my actions generally surprise me.
- I am in full control of what I do.
- The decision whether and when to act is within my hands.
- I am completely responsible for everything that results from my actions.
- Things I do are subject only to my free will.
- While I am in action, I feel like I am a remote controlled robot.
- I am just an instrument in the hands of somebody or something else.
- My movements are automatic—my body simply makes them.
- My behavior is planned by me from the very beginning to the very end.
- My actions just happen without my intention.

Scale: 7-point scale from “strongly disagree” to “strongly agree”

## Self-Efficiacy Scale

In the following, we would like to ask you a few questions about your perception of self-efficacy. To do this, we will use an existing questionnaire on general self-efficacy expectations (Jerusalem & Schwarzer, 1999). Please answer the following questions honestly and to the best of your knowledge.

Please indicate how much you agree with the following statements.

Items (presented in randomized order):

- I can find a solution to every problem.
- I face difficulties calmly because I can always trust my abilities.
- In unexpected situations, I always know how to behave.
- Whatever happens, I'll be fine.
- Even when unexpected events occur, I believe that I can cope with them well.
- When I encounter something new, I know how to deal with it.
- I always succeed in solving difficult problems when I try hard.
- When a problem arises, I can overcome it on my own.
- I have no difficulty achieving my intentions and goals.
- When I encounter resistance, I find ways and means to assert myself.

Scale: 4-point scale: Not true; hardly true; somewhat true; completely true

# Supplementary Analyses

## Supplementary analyses related to Hypothesis 1

### Association of general self-efficacy and mental health

To investigate the relationship between self-efficacy and mental health, the same analyses as those used in the main section on agency experience were conducted (Pearson correlation between participants' self-assessment of their mental health and their mean scores on the General Self-Efficacy Scale; one-way ANOVA (factor: self-reported mental health status; dependent variable: General Self-Efficacy Scale), followed by pairwise independent samples *t*-tests, two-tailed).

The results revealed a highly significant correlation between self-rated overall mental health and the self-efficacy score, $r = .50$, $p < .001$. Thus, higher self-efficacy was linked to better mental health, exceeding the strength of the association found for agency experience. The ANOVA and subsequent t-tests showed patterns similar to those found for the agency experience: The ANOVA revealed a significant main effect of self-reported mental health on the self-efficacy score, $F(2, 209) = 9.94$, $p < .001$, $\eta_p^2 = .09$. The results of the subsequent *t*-tests revealed no significant difference in self-efficacy between individuals with subclinical mental health problems and those without any reported mental health issues, $M_{without} = 2.9$, $n_{without} = 84$, $M_{subclinical} = 2.7$, $n_{subclinical} = 77$, $t(159) = 1.96$, $p = .05$, d = 0.31. Individuals without mental health problems showed significantly higher self-efficacy scores than those with clinically relevant psychological disorders, $M_{clinical} = 2.5$, $n_{clinical} = 51$; $t(133) = 4.12$, $p < .001$, $d = 0.74$. Participants with only subclinical symptoms showed higher self-efficacy scores than those with clinically relevant disorders, $t(126) = 2.79$, $p = .006$, $d = 0.50$ (see Figure S4).

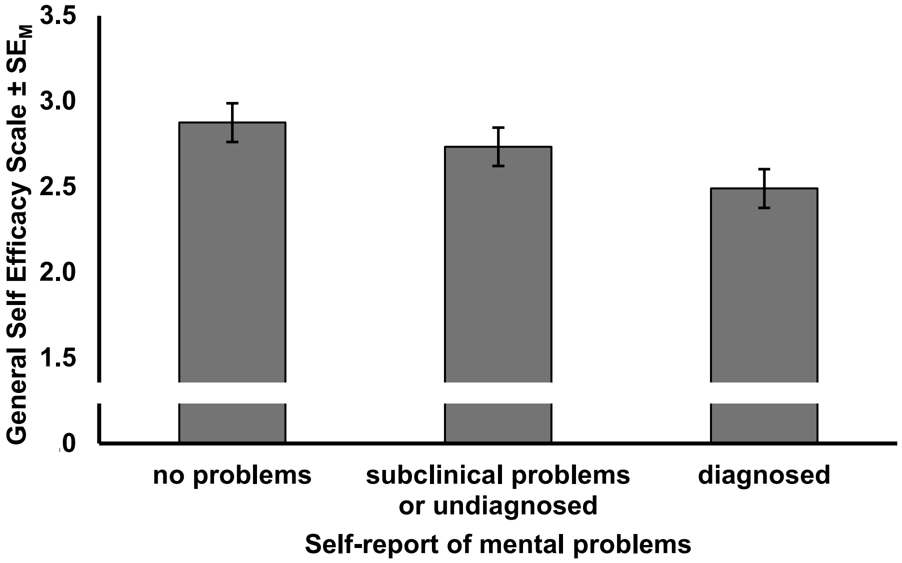


Figure S4. Mean General Self Efficacy Scale scores across different mental health status groups.

## Supplementary analyses related to Hypothesis 2

### Separate regression analyses for personal and global situations

To gain a clearer understanding of the situational influence, separate multiple hierarchical regression analyses were conducted for personally and globally challenging situations. In both models, perceived control, responsibility, and strain served as predictors, while the perceived impact on mental health was entered as the dependent variable.

For personally challenging situations, hierarchical regression analyses revealed that perceived control, responsibility, and strain significantly predicted perceived mental health impacts (see Table S1). While perceived control initially showed only a moderate negative association with adverse mental health outcomes, this effect became more pronounced once perceived responsibility was added to the model. Perceived responsibility, in contrast, was positively related to negative mental health effects. The inclusion of perceived strain further improved the model fit, explaining 47.1% of the variance in perceived mental health outcomes. All three predictors remained significant, with perceived strain emerging as the strongest predictor.

**Table S1.**
*Hierarchical regression analyses predicting mental health for personal challenging situations from perceived control, responsibility, and strain*

| | *Model* | $R^2$ | *Test* | *Predictor* | *Coefficent* |
|---|---|---|---|---|---|
| Step 1 | mental health ~ perceived control | 2,7 % | $F(1,210)$ = 5.80, $p$ = .017 | perceived control | $b$ = - 0.10, $t$ = -2.41, $p$ = .017 |
| Step 2 | mental health ~ perceived control + perceived responsibility | 16.0 % | $F(2,209)$ = 19.87, $p$ < .001 | perceived control | $b$ = - 0.22, $t$ = -4.86, $p$ < .001 |
| | | | | perceived responsibility | $b$ = 0.28, $t$ = 5.75, $p$ < .001 |
| Step 3 | mental health ~ perceived control + perceived responsibility + perceived strain | 47.1 % | $F(3,211)$ = 61.82, $p$ < .001 | Perceived control | $b$ = -0.11, $t$ = -3.07, $p$ = .002 |
| | | | | perceived responsibility | $b$ = 0.10, $t$ = 2.39, $p$ = .018 |
| | | | | perceived strain | $b$ = 0.49, $t$ = 11.07, $p$ < .001 |

For globally challenging situations, a somewhat different pattern emerged (see Table S2). In the first step, perceived control was positively associated with negative mental health effects - contrary to expectations. However, after perceived responsibility was added, the sign of the control coefficient shifted in the expected negative direction, although the effect was no longer significant. Perceived responsibility, in contrast, became a significant positive predictor, indicating that higher perceived responsibility was linked to stronger adverse effects on mental health. With the inclusion of perceived strain in the final model, the explained variance increased substantially to 29.6% with only perceived strain as a significant predictor.

**Table S2.**
*Hierarchical regression analyses predicting mental health for globally challenging situations from perceived control, responsibility, and strain*

| | *Model* | $R^2$ | *Test* | *Predictor* | *Coefficent* |
|---|---|---|---|---|---|
| Step 1 | mental health ~ perceived control | 3.4 % | $F(1,209) = 7.45$, $p = .007$ | perceived control | $b = 0.12$, $t = 2.73$, $p = .007$ |
| Step 2 | mental health ~ perceived control + perceived responsibility | 10.1 % | $F(2,208) = 11.66$, $p < .001$ | perceived control | $b = - 0.03$, $t = -0.50$, $p = .619$ |
| | | | | perceived responsibility | $b = 0.20$, $t = 3.92$, $p < .001$ |
| Step 3 | mental health ~ perceived control + perceived responsibility + perceived strain | 29.6 % | $F(3,207) = 28.95$, $p < .001$ | perceived control | $b = -0.00$, $t = -0.16$, $p = .875$ |
| | | | | perceived responsibility | $b = 0.05$, $t = 0.88$, $p = .378$ |
| | | | | perceived strain | $b = 0.24$, $t = 7.56$, $p < .001$ |

**Separate regression analyses for each emotion**

The results in the main section only partially supported the second hypothesis. Moreover, the preceding ANOVA had revealed differences between the various emotions. Therefore, these were analysed separately to provide deeper insight. Multiple hierarchical regressions were again conducted: In Model 1, situation was entered as a predictor (based on the ANOVA results). In Model 2, the primary variable of interest - perceived control - was added. In Model 3, perceived responsibility was included as a third predictor, given its relation to control. The dependent variables were the perceived intensities of the individual negative emotions (anxiety, anger, shame, guilt, frustration, sadness, helplessness).

Situation alone was a significant predictor only for guilt, shame, and frustration (see Tables S5, S6 and S7). For anxiety, helplessness, sadness, and anger, situation did not significantly predict emotional intensity (see Tables S3, S4, S8 and S9).

When perceived control was added in the second step, it became a significant predictor for anxiety and sadness, showing the expected negative association with emotional intensity. In contrast, for guilt, shame, and frustration, perceived control unexpectedly displayed a significant positive relationship with emotional intensity. For helplessness and anger, neither situation nor control reached significance.

After perceived responsibility was entered as a third predictor, it emerged as a significant positive predictor for all emotions, except for anger (even the final model did not reach significance). For guilt, shame, and frustration, the previously positive effects of perceived control now shifted in the expected negative direction. For anxiety, sadness and helplessness the negative effect of perceived control became significant and stronger.

The strongest effects and the highest proportion of explained variance were observed for guilt, shame, and helplessness, followed by frustration and anxiety, whereas the models for sadness and anger accounted for comparatively little variance.

In addition, an exploratory analysis was conducted with the positive emotion hope as the dependent variable (see Table S10). Situation initially predicted higher hope levels, but this effect disappeared once perceived control was added. Across subsequent models, perceived control remained the only significant predictor, indicating that greater control was consistently associated with stronger feelings of hope, whereas responsibility showed no effect.

**Table S3.**
*Hierarchical regression analyses predicting anxiety from situation, perceived control, and perceived responsibility*

| | *Model* | $R^2$ | *Test* | *Predictor* | *Coefficent* |
|---|---|---|---|---|---|
| Step 1 | anxiety ~ situation | 0.2 % | $F(1,843) = 1.98$, $p = .159$ | situation | $b = -1.20$, $t = -1.41$, $p = .159$ |
| Step 2 | anxiety ~ situation + perceived control | 1.0 % | $F(2,842) = 4.24$, $p = .015$ | situation | $b = 0.44$, $t = 0.41$, $p = .683$ |
| | | | | perceived control | $b = 0.10$, $t = 2.55$, $p = .011$ |
| Step 3 | anxiety ~ situation + perceived control + perceived responsibility | 15.1 % | $F(3,841) = 49.75$, $p < .001$ | situation | $b = 5.11$, $t = 4.81$, $p < .001$ |
| | | | | perceived control | $b = -0.22$, $t = -4.74$, $p < .001$ |
| | | | | perceived responsibility | $b = 0.52$, $t = 11.81$, $p < .001$ |

**Table S4.**
*Hierarchical regression analyses predicting anger from situation, perceived control, and perceived responsibility*

| | *Model* | $R^2$ | *Test* | *Predictor* | *Coefficent* |
|---|---|---|---|---|---|
| Step 1 | anger ~ situation | 0.2 % | $F(1,843) = 1.37$, $p = .243$ | situation | $b = -0.93$, $t = -1.17$, $p = .243$ |
| Step 2 | anger ~ situation + perceived control | 0.2% | $F(2,842) = 0.80$, $p = .448$ | situation | $b = -0.63$, $t = -0.63$, $p = .526$ |
| | | | | perceived control | $b = 0.02$, $t = 0.49$, $p = .622$ |
| Step 3 | anger ~ situation + perceived control + perceived responsibility | 0.4 % | $F(3,841) = 1.043$, $p = .373$ | situation | $b = -0.14$, $t = -0.13$, $p = .897$ |
| | | | | perceived control | $b = -0.02$, $t = -0.33$, $p = .742$ |
| | | | | perceived responsibility | $b = 0.06$, $t = 1.23$, $p = .218$ |

**Table S5.**
*Hierarchical regression analyses predicting shame from situation, perceived control, and perceived responsibility*

| | *Model* | $R^2$ | *Test* | *Predictor* | *Coefficent* |
|---|---|---|---|---|---|
| Step 1 | shame ~ situation | 6.7 % | $F$(1,842) = 60.77, $p$ < .001 | situation | $b$ = -6.40, $t$ = -7.80, $p$ < .001 |
| Step 2 | shame ~ situation + perceived control | 9.2 % | $F$(2,841) = 42.36, $p$ < .001 | situation | $b$ = -3.51, $t$ = -3.46, $p$ < .001 |
| | | | | perceived control | $b$ = 0.18, $t$ = 4.73, $p$ < .001 |
| Step 3 | shame ~ situation + perceived control + perceived responsibility | 29.6 % | $F$(3,840) = 117,94, $p$ < .001 | situation | $b$ = 2.11, $t$ = 2.19, $p$ = .029 |
| | | | | perceived control | $b$ = -0.20, $t$ = -4.90, $p$ < .001 |
| | | | | perceived responsibility | $b$ = 0.63, $t$ = 15.64, $p$ < .001 |

**Table S6.**
*Hierarchical regression analyses predicting guilt from situation, perceived control, and perceived responsibility*

| | *Model* | $R^2$ | *Test* | *Predictor* | *Coefficent* |
|---|---|---|---|---|---|
| Step 1 | guilt ~ situation | 20.5 % | $F$(1,842) = 216.87, $p$ < .001 | situation | $b$ = -11.59, $t$ = -14.73, $p$ < .001 |
| Step 2 | guilt ~ situation + perceived control | 26.7 % | $F$(2,841) = 152.99, $p$ < .001 | situation | $b$ = -6.78, $t$ = -7.17, $p$ < .001 |
| | | | | perceived control | $b$ = 0.30, $t$ = 8.43, $p$ < .001 |
| Step 3 | guilt ~ situation + perceived control + perceived responsibility | 50.1 % | $F$(3,840) = 281.31, $p$ < .001 | situation | $b$ = -0.54, $t$ = -0.65, $p$ = .519 |
| | | | | perceived control | $b$ = -0.13 $t$ = -3.50, $p$ < .001 |
| | | | | perceived responsibility | $b$ = 0.70, $t$ = 19.87, $p$ < .001 |

**Table S7.**
*Hierarchical regression analyses predicting frustration from situation, perceived control, and perceived responsibility*

| | Model | $R^2$ | Test | Predictor | Coefficent |
|---|---|---|---|---|---|
| Step 1 | frustration ~ situation | 10.0 % | $F(1,843) = 94.06$, $p < .001$ | situation | $b = -7.10$, $t = -9.70$, $p < .001$ |
| Step 2 | frustration ~ situation + perceived control | 10.7 % | $F(2,842) = 50.18$, $p < .001$ | situation | $b = -5.77$, $t = -6.28$, $p < .001$ |
| | | | | perceived control | $b = 0.08$, $t = 2.40$, $p = .016$ |
| Step 3 | frustration ~ situation + perceived control + perceived responsibility | 17.3 % | $F(3,841) = 58.53$, $p < .001$ | situation | $b = -2.90$, $t = -3.05$, $p = .002$ |
| | | | | perceived control | $b = -0.12$, $t = -2.85$, $p = .004$ |
| | | | | perceived responsibility | $b = 0.33$, $t = 8.21$, $p < .001$ |

**Table S8.**
*Hierarchical regression analyses predicting sadness from situation, perceived control, and perceived responsibility*

| | Model | $R^2$ | Test | Predictor | Coefficent |
|---|---|---|---|---|---|
| Step 1 | sadness ~ situation | 0.2 % | $F(1,844) = 1.71$, $p = .191$ | situation | $b = -1.11$, $t = -1.31$, $p = .191$ |
| Step 2 | sadness ~ situation + perceived control | 0.8 % | $F(2,843) = 3.39$, $p = .034$ | situation | $b = -2.56$, $t = -2.40$, $p = .016$ |
| | | | | perceived control | $b = -0.09$, $t = -2.25$, $p = .025$ |
| Step 3 | sadness ~ situation + perceived control + perceived responsibility | 4.0 % | $F(3,842) = 11.70$, $p < .001$ | situation | $b = -0.33$, $t = -0.29$, $p = .769$ |
| | | | | perceived control | $b = -0.24$, $t = -4.98$, $p < .001$ |
| | | | | perceived responsibility | $b = 0.25$, $t = 5.30$, $p < .001$ |

**Table S9.**
*Hierarchical regression analyses predicting helplessness from situation, perceived control, and perceived responsibility*

| | *Model* | *$R^2$* | *Test* | *Predictor* | *Coefficent* |
|---|---|---|---|---|---|
| Step 1 | helplessness ~ situation | 0.0 % | $F(1,844) = 0.01$, $p = .911$ | situation | $b = -0.090$, $t = -0.11$, $p = .911$ |
| Step 2 | helplessness ~ situation + perceived control | 0.1 % | $F(2,843) = 0.25$, $p = .777$ | situation | $b = -0.52$, $t = -0.51$, $p = .608$ |
| | | | | perceived control | $b = -0.03$, $t = -0.70$, $p = .483$ |
| Step 3 | helplessness ~ situation + perceived control + perceived responsibility | 10.6 % | $F(3,842) = 33.24$, $p < .001$ | situation | $b = 3.29$, $t = 3.20$, $p < .001$ |
| | | | | perceived control | $b = -0.29$, $t = -6.48$, $p < .001$ |
| | | | | perceived responsibility | $b = 0.43$, $t = 9.96$, $p < .001$ |

**Table S10.**
*Hierarchical regression analyses predicting hope from situation, perceived control, and perceived responsibility*

| | *Model* | *$R^2$* | *Test* | *Predictor* | *Coefficent* |
|---|---|---|---|---|---|
| Step 1 | hope ~ situation | 2.0 % | $F(1,836) = 17.37$, $p < .001$ | situation | $b = -2.89$, $t = -4.17$, $p < .001$ |
| Step 2 | hope ~ situation + perceived control | 7.6 % | $F(2,835) = 34.51$, $p < .001$ | situation | $b = 0.69$, $t = 0.82$, $p = .413$ |
| | | | | perceived control | $b = 0.22$, $t = 7.12$, $p < .001$ |
| Step 3 | hope ~ situation + perceived control + perceived responsibility | 7.7 % | $F(3,834) = 23.09$, $p < .001$ | situation | $b = 0.50$, $t = 0.55$, $p = .581$ |
| | | | | perceived control | $b = 0.24$, $t = 6.04$, $p < .001$ |
| | | | | perceived responsibility | $b = -0.02$, $t = -0.55$, $p = .583$ |

**Regression analyses on the general perception of challenging situations**

Similar to the concrete situations presented in the main section, exploratory multiple hierarchical regressions were conducted for the more general assessment of stressful personal and global situations. In this case, perceived control, perceived overwhelm, thought frequency, and distress were entered stepwise as predictors. The perceived effects on mental health again served as the dependent variable.

For general perception of personally challenging situations, perceived control initially showed a protective association with mental health, suggesting that greater control was linked to fewer negative effects (see Table S11). When perceived overwhelm and thought frequency were added, both emerged as significant positive predictors, whereas the effect of control temporarily disappeared. In the final model, perceived strain became the strongest predictor of adverse mental health outcomes, while perceived control regained its significant negative association.

**Table S11.**
*Hierarchical regression analyses predicting mental health for personal challenging situations from perceived control, overwhelm, thought frequency, and perceived strain*

| | *Model* | *$R^2$* | *Test* | *Predictor* | *Coefficent* |
|---|---|---|---|---|---|
| Step 1 | mental health ~ perceived control | 7.0 % | $F(1,204) = 15.07$, $p < .001$ | perceived control | $b = -0.19$, $t = -3.88$, $p < .001$ |
| Step 2 | mental health ~ perceived control + perceived overwhelm | 32.3 % | $F(2,203) = 48.44$, $p < .001$ | perceived control | $b = -0.05$, $t = -1.10$, $p = .273$ |
| | | | | perceived overwhelm | $b = 0.31$, $t = 8.73$, $p < .001$ |
| Step 3 | mental health ~ perceived control + perceived overwhelm + thought frequency | 35.6 % | $F(3,202) = 37.24$, $p < .001$ | perceived control | $b = -0.08$, $t = -1.72$, $p = .087$ |
| | | | | perceived overwhelm | $b = 0.20$, $t = 4.28$, $p < .001$ |
| | | | | thought frequency | $b = 0.17$, $t = 3.22$, $p < .001$ |
| Step 4 | mental health ~ perceived control + perceived overwhelm + thought frequency + perceived strain | 60.9 % | $F(4,201) = 78.14$, $p < .001$ | perceived control | $b = -0.14$, $t = -4.02$, $p < .001$ |
| | | | | perceived overwhelm | $b = 0.04$ $t = 1.13$, $p = .260$ |
| | | | | thought frequency | $b = 0.12$, $t = 2.82$, $p = .005$ |
| | | | | perceived strain | $b = 0.50$ $t = 11.39$, $p < .001$ |

For globally challenging situations, perceived control initially showed a small positive association with negative mental health outcomes, which disappeared once perceived overwhelm was added (see Table S12). Overwhelm and thought frequency then emerged as

significant predictors. After the inclusion of perceived strain, it remained the only significant predictor.

**Table S12.**
*Hierarchical regression analyses predicting mental health for global challenging situations from perceived control, overwhelm, thought frequency, and perceived strain*

| | *Model* | $R^2$ | *Test* | *Predictor* | *Coefficent* |
|---|---|---|---|---|---|
| Step 1 | mental health ~ perceived control | 2.6 % | $F(1,206) = 5.54$, $p = .020$ | perceived control | $b = 0.12$, $t = 2.35$, $p = .020$ |
| Step 2 | mental health ~ perceived control + perceived overwhelm | 29.3 % | $F(2,205) = 42.46$, $p < .001$ | perceived control | $b = 0.05$, $t = 1.09$, $p = .277$ |
| | | | | perceived overwhelm | $b = 0.29$, $t = 8.79$, $p < .001$ |
| Step 3 | mental health ~ perceived control + perceived overwhelm + thought frequency | 31.9 % | $F(3,204) = 31.85$, $p < .001$ | perceived control | $b = 0.03$, $t = 0.76$, $p = .447$ |
| | | | | perceived overwhelm | $B = 0.20$, $t = 4.22$, $p < .001$ |
| | | | | thought frequency | $b = 0.14$, $t = 2.80$, $p = .006$ |
| Step 4 | mental health ~ perceived control + perceived overwhelm + thought frequency + perceived strain | 37.5 % | $F(4,203) = 30.48$, $p < .001$ | perceived control | $b = 0.01$, $t = 0.26$, $p = .798$ |
| | | | | perceived overwhelm | $b = 0.11$, $t = 2.28$, $p = .024$ |
| | | | | thought frequency | $b = 0.10$, $t = 2.00$, $p = .046$ |
| | | | | perceived strain | $b = 0.21$ $t = 4.28$, $p < .001$ |

## Supplementary analyses related to Hypothesis 3

### Relationship between trait sense of agency and the perceived helpfulness of coping strategies

Exploratory analyses were further conducted to complement the third hypothesis (which examined the relationship between trait sense of agency and the frequency of using different coping strategies). These analyses explored the association between trait sense of agency and the *perceived helpfulness* of the coping strategies. To this end, Pearson correlations were computed between the scores of the Sense of Agency Scale and the reported perceived helpfulness of the different coping strategies.

Significant correlations emerged between the Sense of Agency Scale (SoAS) scores and the strategies *Acceptance*, $r = .27$, $p < .001$, and *Optimism*, $r = .30$, $p < .001$. Additionally, a small positive correlation was found with *Knowledge*, $r = .18$, $p = .010$, and the strongest positive correlation was observed with *Focus on Taking Actions*, $r = .34$, $p < .001$. Overall, the pattern of correlations was similar to that observed for the *frequency of use* of coping strategies, although slightly more pronounced in this case.

### Relationship between General Self-Efficacy and coping

To investigate the association of general self-efficacy and coping, correlations were conducted between General Self-Efficacy Scores and ratings of perceived helpfulness as well as the use of different coping strategies.

Results revealed significant correlations of self-efficacy scores with the helpfulness-ratings of *Acceptance*, $r = .15$, $p =.031$, *Optimism*, $r = .29$, $p < .001$, *Giving Up Responsibility*, $r = -.14$, $p = .038$, *Knowledge,* $r = .19$, $p = .006$, *Focus on Taking Actions*, $r = .30$, p < .001.

For self-efficacy scores and use of coping strategies significant correlations emerged for all strategies except of *Professional Support*; *Optimism,* $r = .41$, $p <.001$; *Focus on Taking Action*, $r = .31$, $p <.001$; *Avoidance & Distraction*, $r = -.28$, $p <.001$; *Acceptance,* $r = .20$, $p =.003$; *Giving Up Responsibility*, $r = -.17$, $p = .014$; *Social Support*, $r = .17$, $p = .016$; *Knowledge*, $r = .14$, $p = .039$.

## Supplementary analyses related to Hypothesis 4

### Discrepancy between Use and Helpfulness of coping strategies

A further exploratory analysis compared the *frequency of use* and the *perceived helpfulness* of the coping strategies. The repeated-measures ANOVA conducted as part of the fourth hypothesis revealed a significant main effect for the evaluation aspect (frequency of use vs. perceived helpfulness), $F(1, 184) = 70.88$, $p < .001$, $\eta p^2 = .28$ (see Figure 5 in the main manuscript). Follow-up *t*-tests for dependent samples were conducted to explore these relationships in greater detail.

The results showed significant differences between frequency of use and perceived helpfulness for all eight coping strategies. For all strategies except *Avoidance & Distraction*, perceived helpfulness ratings were significantly higher than ratings of frequency of use. The largest difference was found for the strategy *Professional Support*, $M_{use} = 29.8$, $M_{helpfulness} = 64.5$, $t(187) = –13.98$, $p < .001$, $d_z = –1.02$. The second-largest difference was observed for *Focus on Taking Actions*, $M_{use} = 64.3$, $M_{helpfulness} = 77.6$, $t(210) = –7.38$, $p < .001$, $d_z = –0.51$. The remaining strategies also showed significant differences in the same direction, though with smaller effect sizes: *Social Support*, $M_{use} = 62.0$, $M_{helpfulness} = 73.0$, $t(209) = –5.88$, $p < .001$, $d_z = –0.41$; *Optimism*, $M_{use} = 49.9$, $M_{helpfulness} = 57.9$, $t(211) = –5.02$, $p < .001$, $d_z = –0.34$; *Knowledge*, $M_{use} = 57.7$, $M_{helpfulness} = 65.8$, $t(207) = –4.75$, $p < .001$, $d_z = –0.33$; *Giving Up Responsibility*, $M_{use} = 32.9$, $M_{helpfulness} = 40.2$, $t(206) = –3.69$, $p < .001$, $d_z = –0.26$; *Acceptance*, $M_{use} = 57.9$, $M_{helpfulness} = 63.8$, $t(211) = –2.98$, $p = .003$, $d_z = –0.20$.

An exception to this pattern was the strategy *Avoidance & Distraction*, which showed a reversed pattern, $M_{use} = 65.0$, $M_{helpfulness} = 41.2$, $n = 212$, $t(211) = 10.32$, $p < .001$, $d_z = 0.71$. This moderate effect indicates that *Avoidance & Distraction* was used frequently yet perceived as relatively unhelpful in comparison.

### Association of the discrepancy between use and helpfulness of coping strategies with trait agency experience

To examine whether the discrepancies between reported use and perceived helpfulness reported above were associated with agency experience, the absolute values of these discrepancies were first calculated. These absolute discrepancy scores were then correlated with the Sense of Agency Scale (SoAS). This was done both for each individual coping strategy as well as for the mean discrepancy across all strategies.

The results of the analysis using the mean discrepancy across strategies indicate that a higher perceived sense of agency is associated with a lower discrepancy between reported use and perceived helpfulness of coping strategies, $r = –.20$, $p = .004$. When examining individual strategies, this effect emerged only for the strategies *Avoidance and Distraction* ($r = –.15$, $p = .027$), *Social Support* ($r = –.19$, $p = .005$), and *Focusing on Taking Action* ($r = –.20$, $p = .003$).

## Analysis of open questions regarding mental health

A descriptive analysis was conducted on participants' responses to the open-ended questions regarding mental health, to further describe the sample.

In total, 103 meaningful responses were provided to the question regarding *subclinical mental health problems*. The most frequently mentioned issues were **stress** (50 mentions), **subclinical depressive symptoms** (52 mentions), and **various forms of anxiety** (19 mentions). Other, less frequently reported difficulties included problems related to motivation, drive, and self-esteem.

The question concerning *clinically diagnosed mental disorders* was meaningfully answered by 47 participants. **Depression** (39 mentions) was by far the most frequently reported disorder, followed by **anxiety disorders** (12 mentions). Less frequently mentioned conditions included adjustment disorders, eating disorders, ADHD, borderline personality disorder, PTSD, and, in rare cases, obsessive-compulsive disorder.

A total of 76 participants provided information regarding the *use of professional mental health services*. The most frequently utilized forms of support were **psychotherapy** (58 mentions) and **medication** (29 mentions). Additional reported sources of help included visits to doctors, hospital stay, counseling centers, as well as coaching and mindfulness courses.
